# The ABA receptor NtPYL6 promotes flavonol biosynthesis to enhance tobacco resistance to UV-B

**DOI:** 10.1093/plphys/kiag257

**Published:** 2026-04-29

**Authors:** Zhong Wang, Li Xu, Haitao Huang, Yali Liu, Pingping Liu, Jiarui Jiang, Xin Xu, Huina Zhou, Qiansi Chen, Xuemei Li, Qian Gao, Jun Yang

**Affiliations:** China Tobacco Gene Research Center, Zhengzhou Tobacco Research Institute of CNTC, Zhengzhou 450001, China; Yunnan Key Laboratory of Tobacco Chemistry, R&D Center of China Tobacco Yunnan Industrial Co. Ltd., Kunming 650202, China; Yunnan Key Laboratory of Tobacco Chemistry, R&D Center of China Tobacco Yunnan Industrial Co. Ltd., Kunming 650202, China; China Tobacco Gene Research Center, Zhengzhou Tobacco Research Institute of CNTC, Zhengzhou 450001, China; China Tobacco Gene Research Center, Zhengzhou Tobacco Research Institute of CNTC, Zhengzhou 450001, China; Yunnan Key Laboratory of Tobacco Chemistry, R&D Center of China Tobacco Yunnan Industrial Co. Ltd., Kunming 650202, China; China Tobacco Gene Research Center, Zhengzhou Tobacco Research Institute of CNTC, Zhengzhou 450001, China; China Tobacco Gene Research Center, Zhengzhou Tobacco Research Institute of CNTC, Zhengzhou 450001, China; China Tobacco Gene Research Center, Zhengzhou Tobacco Research Institute of CNTC, Zhengzhou 450001, China; Yunnan Key Laboratory of Tobacco Chemistry, R&D Center of China Tobacco Yunnan Industrial Co. Ltd., Kunming 650202, China; Yunnan Key Laboratory of Tobacco Chemistry, R&D Center of China Tobacco Yunnan Industrial Co. Ltd., Kunming 650202, China; China Tobacco Gene Research Center, Zhengzhou Tobacco Research Institute of CNTC, Zhengzhou 450001, China

## Abstract

ABA and flavonol accumulation protect plants against UV-B radiation damage. However, the molecular mechanism by which UV-B enhances ABA signaling to induce flavonol biosynthesis remains largely unknown. Here, we found that ABA receptor *ntpyl6* (PYRABACTIN RESISTANCE 1-LIKE6) mutants are more sensitive to UV-B than the wild type and that UV-B significantly induces ABA accumulation and *NtPYL6* expression in tobacco (*Nicotiana tabacum*). The induction of UV-B on *NtPYL6* expression was achieved by inactivating NtBES1 (BRI1-EMS-SUPPRESSOR1), which directly binds to the E-box present in the *NtPYL6* promoter to inhibit its expression. Metabolome and transcriptome analyses demonstrated that NtPYL6 positively mediates flavonol accumulation and the expression of related genes induced by ABA signaling. We further showed that NtPYL6-NtABI1/NtHAB1-NtSnRK2.12 constitute the core ABA signaling pathway to activate NtABF3 (ABA-RESPONSIVE ELEMENT-BINDING FACTOR 3). NtABF3 directly binds to the ABRE cis-elements present in the promoters of *NtCHS* and *NtFLS* genes to induce their transcription, leading to the increased accumulation of flavonols and enhanced plant resistance to UV-B. Our results elucidate a mechanism by which enhanced ABA signaling promotes flavonol accumulation, thereby improving plant resistance to UV-B.

## Teaser

ABA signaling enhances plant resistance to UV-B stress by inducing flavonol biosynthesis, which enriches the understanding and provides insights for improvement of plant responses to UV-B stress.

## Introduction

Plants have evolved efficient mechanisms to protect themselves from the damage caused by UV-B (280 to 315 nm) light. One important strategy is to enhance the accumulation of flavonoids, mainly including flavonols, anthocyanins, and proanthocyanins, which can help plants scavenge the reactive oxygen species (ROS) caused by UV-B, thereby preventing or limiting its damage (Li et al. 1993; Agati and Tattini 2010; Clayton et al. 2018; Liang et al. 2020). UV-B induces monomerization and nuclear accumulation of its photoreceptor UVRESISTANCE LOCUS 8 (UVR8) (Kaiserli and Jenkins 2007; Rizzini et al. 2011). The nucleus-activated UVR8 interacts with BRI1-EMS-SUPPRESSOR1 (BES1) to remove its direct inhibition on the expression of *MYB11*, *MYB12*, and *MYB111*, which are well-known promoters of flavonols biosynthesis (Stracke et al. 2007; Liang et al. 2018, 2020). BES1 has also been shown to induce the expression of *MYB27* to modulate the flavonols synthesis in response to UV-B in tobacco (*Nicotiana tabacum*) (Wang et al. 2024). In addition, UV-B integrates various plant hormones to modulate the balance between plant growth and photoprotection responses (Vanhaelewyn et al. 2016). The abscisic acid (ABA) accumulation and signaling have been shown to be enhanced by UV-B stress in plants (Peng and Zhou 2009; Tossi et al. 2009; Ðinh et al. 2013; Pan et al. 2014; Shi et al. 2023), and exogenous ABA alleviates the UV-B damage in *Rhododendron chrysanthum* (Yu et al. 2024). However, the mechanism by which enhanced ABA signaling contributes to plant resistance to UV-B radiation still needs to be further clarified.

Exogenous ABA can enhance the accumulation of both anthocyanins and flavonols in plants, and its induction degree on flavonols could even be much greater than that of anthocyanins (Loreti et al. 2008; Berli et al. 2010, 2011; Lai et al. 2014, 2019; An et al. 2021; Han et al. 2021). ABA binds to the PYRABACTIN RESISTANCE/PYR1-LIKE/REGULATORY COMPONENTS OF THE ABA RECEPTOR its receptors (PYR/PYLs/RCAR, hereafter PYLs) to enhance their combination with the PROTEIN PHOSPHATASE 2C (PP2C) proteins removing the inhibition of PP2Cs on SNF1-RELATED PROTEIN KINASE 2 (SnRK2). SnRK2 phosphorylates and activates ABA-RESPONSIVE ELEMENT BINDING PROTEIN/ABA-RESPONSIVE ELEMENT BINDING FACTORS (AREB/ABFs, hereafter ABFs) to modulate the downstream ABA-responsive genes (Fujii et al. 2009; Park et al. 2009; Zhu 2016). The ABF transcription factor ABSCISIC ACID-INSENSITIVE5 (ABI5) induces and interacts with bHLH3 to enhance its binding to *DIHYDROFLAVONOL 4-REDUCTASE* (*DFR*) and *UDP FLAVONOID GLUCOSYL TRANSFERASE* (*UF3GT*) genes, thus promoting the accumulation of anthocyanins in apples (*Malus domestica*) (An et al. 2021). ABI5 is also suggested to mediate the ABA-induced flavonols synthesis by interacting with ELONGATED HYPOCOTYL5 (HY5), which further targets *MYB12* and *MYB111* to induce the accumulation of flavonols (Brunetti et al. 2019). This hypothesis still needs to be further verified, and there is still a lack of understanding concerning the regulation of ABA signaling on flavonols biosynthesis.

Multi-omics analysis in *R. chrysanthum* suggests that exogenous ABA mitigates the detrimental effects of UV-B possibly by activating the phenylpropanoid biosynthesis pathway, which produces flavonoids and other antioxidants (Yu et al. 2025). In soybean suspension cells, UV-B triggers an ABA signal that further promotes the accumulation of isoflavones to cope with UV-B damage (Wang et al. 2025). Although several omics clues suggest that ABA might enhance plant resistance to UV-B by regulating the synthesis of flavonoids, the details of the implementation mechanism of this inference still needs further clarification. Here, we show that UV-B relieves the inhibition of NtBES1 on *NtPYL6* expression. NtPYL6 mediates the ABA-enhanced UV-B resistance in tobacco. We further demonstrate that ABA-NtPYL6 signaling pathway activates the NtABF3, which directly induced the expression of *NtCHS* and *NtFLS* genes to promote the synthesis of flavonols, thereby enhancing tobacco resistance to UV-B. Our results reveal the detailed regulatory mechanism of the NtPYL6-mediated ABA signal in enhancing plant UV-B resistance by inducing flavonol accumulation.

## Results

### Knock-out of *NtPYL6* enhances the sensitivity of tobacco plants to UV-B

**Figure 1:**
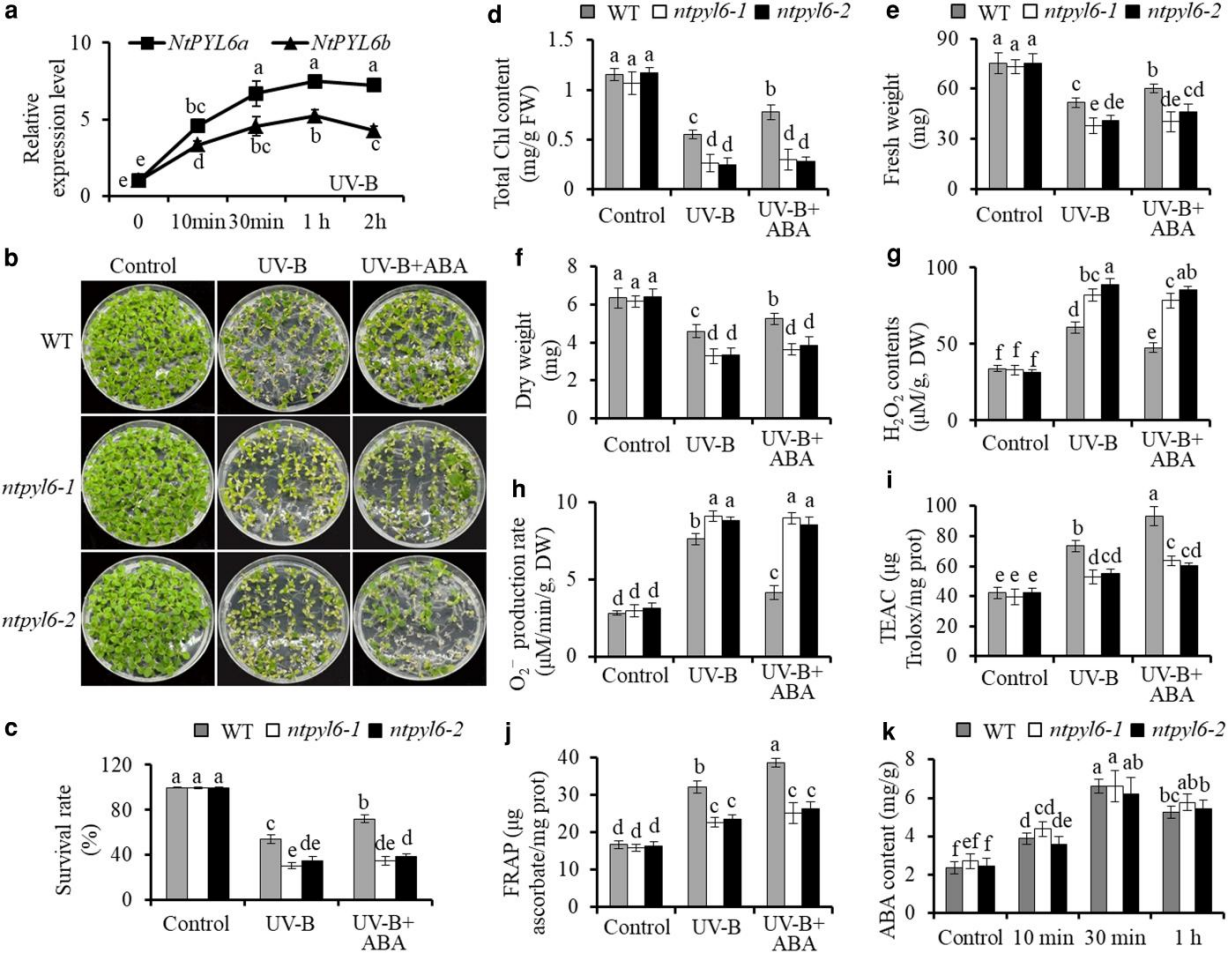
*Ntpyl6* mutants are more sensitive to UV-B radiation than WT. a) Relative expression levels of *NtPYL6a* and *NtPYL6b* genes in WT plants treated with UV-B for different times. Values are means ± SD of 3 independent replicates. Tukey test was performed to detect the significant differences among different samples (*P* < 0.05), which were shown with letters. b) Phenotypes of WT, *ntpyl6-1*, and *ntpyl6-2* seedlings with or without UV-B treatment. ABA, abscisic acid. c) Survival rates of the control and UV-B–treated WT, *ntpyl6-1*, and *ntpyl6-2* seedlings. Values are means ± SD of 5 independent replicates. Tukey test was performed to detect the significant differences among different samples (*P* < 0.05), which were shown with letters. d) Total Chl contents in the WT, *ntpyl6-1*, and *ntpyl6-2* seedlings under normal or UV-B treatment. FW, fresh weight. Values are means ± SD of 5 independent replicates. Tukey test was performed to detect the significant differences among different samples (*P* < 0.05), which were shown with letters. e, f) Fresh and dry weight of the above-ground parts of WT, *ntpyl6-1*, and *ntpyl6-2* seedlings. Ten seedlings were weighed together as a replicate, and the values were exhibited as the weight per seedling. Values are means ± SD of 10 independent replicates. Tukey test was performed to detect the significant differences among different samples (*P* < 0.05), which were shown with letters. g, h) The contents of H_2_O_2_ and O_2_^−^ in the WT, *ntpyl6-1*, and *ntpyl6-2* seedlings under different treatments. Abbreviation: DW, dry weight. Values are means ± SD of 5 independent replicates. Tukey test was performed to detect the significant differences among different samples (*P* < 0.05), which were shown with letters. i, j) Total antioxidant capacities of the WT, *ntpyl6-1*, and *ntpyl6-2* seedlings under different treatments. Abbreviations: FRAP, ferric reducing capacity; TEAC, trolox equivalent antioxidant capacity. Values are means ± SD of 5 independent replicates. Tukey test was performed to detect the significant differences among different samples (*P* < 0.05), which were shown with letters. k) Measurement of ABA contents in WT, *ntpyl6-1*, and *ntpyl6-2* seedlings treated with UV-B for different time. Values are means ± SD of 3 independent replicates. Tukey test was performed to detect the significant differences among different samples (*P* < 0.05), which were shown with letters.

ABA receptors in tobacco are encoded by 29 putative *PYL* genes, among which *NtPYL6* and *NtPYL7* are close homologous genes and suggested to play pivotal roles in response to abiotic stresses (Bai et al. 2019). We then cloned these 2 genes and renamed them *NtPYL6a* and *NtPYL6b*, respectively, since they shared 94.55% identity at the genomic level, 94.06% identity at the amino acid level, and the same exon/intron structures (Fig. S1a-c). To verify whether the transcription of *NtPYL6* is induced in response to UV-B, we detected the expression levels of *NtPYL6a* and *NtPYL6b* in tobacco seedlings treated with UV-B radiation. Compared with the control, the expression level of *NtPYL6a* was significantly upregulated by 3.6-, 5.7-, 6.5-, and 6.2-fold in the seedlings treated with UV-B for 10 min, 30 min, 1 h, and 2 h (Fig. 1a), respectively. The expression level of *NtPYL6b* also significantly increased by 2.3-, 3.5-, 4.2-, and 3.3-fold, respectively, in these UV-B treated seedlings (Fig. 1a), suggesting that UV-B induced the expression of *NtPYL6* gene. Two independent *ntpyl6* lines were obtained, 1 (*ntpyl6-1*) of which had a deletion of A in both *NtPYL6a* and *NtPYL6b*, while the other (*ntpyl6-2*) had a deletion of T in the 2 genes (Fig. S1d). Both deletions disturbed the open reading frame (ORF) of *NtPYL6a* and *NtPYL6b* and resulted in truncated proteins (Fig. S1e). Gene expression and protein abundance analysis further confirmed that the transcription and protein coding of *NtPYL6a* and *NtPYL6b* genes were prematurely terminated in the 2 *ntpyl6* mutants (Fig. S1f-g). The *ntpyl6-1* and *ntpyl6-2* mutants showed reduced sensitivity to ABA treatment compared with wild type (WT) (Fig. S1h-i), suggesting that NtPYL6 mediates the ABA signal transduction in tobacco.

The WT and *ntpyl6* seedlings were then treated with UV-B radiation to test whether NtPYL6 is involved in plant response to UV-B. WT, *ntpyl6-1*, and *ntpyl6-2* seedlings showed no obvious differences under normal conditions. However, the survival rate of WT seedlings significantly reduced to 53.6% after UV-B treatment, and those of *ntpyl6-1* and *ntpyl6-2* were 30.3% and 34.4%, respectively, which were both significantly lower than that of WT (Fig. 1b,1c). The chlorophyll (Chl) contents in WT, *ntpyl6-1*, and *ntpyl6-2* leaves showed no significant differences under normal conditions, but the Chl contents in *ntpyl6-1* and *ntpyl6-2* leaves treated with UV-B were separately 52.5% and 55.4% lower than that of UV-B treated WT leaves (Fig. 1d). Consistently, the fresh and dry weight of UV-B–treated *ntpyl6-1* and *ntpyl6-2* plants were all significantly lower than that of WT plants treated with UV-B (Fig. 1e, 1f). The accumulation of H_2_O_2_ and O_2_^−^ significantly increased by 79.3% and 169.8%, respectively, in WT plants treated with UV-B compared with that under normal conditions, while the H_2_O_2_ and O_2_^−^ contents in UV-treated *ntpyl6-1* and *ntpyl6-2* seedlings were even significantly higher than that in WT seedlings treated with UV-B (Fig. 1g, 1h). In contrast, the antioxidant capacities in UV-B–treated *ntpyl6-1* and *ntpyl6-2* seedlings (measured as TEAC and FRAP) were significantly lower than that of UV-B treated WT plants (Fig. 1i and j). These results indicate NtPYL6 is involved in plant response to UV-B.

Next, we tested whether NtPYL6-mediated UV-B response depends on the ABA signal transduction. Compared with those treated with UV-B alone, the WT seedlings treated with UV-B + ABA showed a significantly higher survival rate, Chl content, fresh/dry weight, and antioxidant capacities but significant lower H_2_O_2_ and O_2_^−^ contents (Fig. 1b-j), indicating that ABA can relieve the damage caused by UV-B in tobacco. However, the survival rate, Chl content, fresh/dry weight, antioxidant capacities, and H_2_O_2_ and O_2_^−^ contents in *ntpyl6-1* and *ntpyl6-2* seedlings treated with UV-B + ABA showed no significant differences from those in UV-B–treated seedlings (Fig. 1b-j), respectively. Compared with the control, the ABA content in WT seedlings treated with UV-B for 10 min, 30 min, and 1 h significantly increased by 62.2%, 179.8%, and 122.2%, respectively, and the contents of ABA in *ntpyl6-1* and *ntpyl6-2* seedlings showed no significant differences from that in WT seedlings under normal or UV-B treatments (Fig. 1k), suggesting that loss of function of NtPYL6 did not affect the UV-B–induced ABA accumulation. Taken together, these results indicate that NtPYL6 might confer plant resistance to UV-B by mediating ABA signaling transduction in tobacco.

### NtBES1 directly inhibits the expression of *NtPYL6* genes

**Figure 2:**
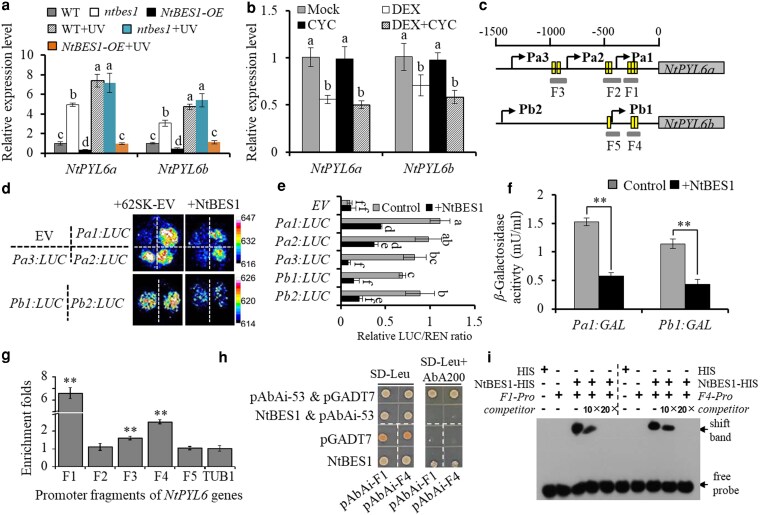
NtBES1 directly inhibits the expression of *NtPYL6* gene. a) Relative expression levels of *NtPYL6a* and *NtPYL6b* genes in the WT, *NtBES1-OE*, and *ntbes1* plants treated with control or UV-B. Values are means ± SD of 3 independent replicates. Tukey test was performed to detect the significant differences among different samples (*P* < 0.05), which were shown with letters. b) Relative expression levels of *NtPYL6a* and *NtPYL6b* genes in the *35S:NtBES1-GR* seedlings under different treatments. Abbreviations: CYC, cycloheximide; DEX, dexamethasone. Values are means ± SD of 3 independent replicates. Tukey's test was performed to detect the significant differences among different samples (*P* < 0.05), which were shown with letters. c) Analyses of the putative promoter sequences of *NtPYL6a* and *NtPYL6b*. Yellow boxes represent the E-box (CANNTG) cis-elements. Pa1-3 and Pb1-2 indicate the fragments used for LUC and *β-GAL* activity analyses. F1-5 shows the fragments detected in the ChIP-qPCR analysis. d) Luciferase (LUC) fluorescence of different reporter vectors constructed with different promoter fragments of *NtPYL6a* and *NtPYL6b*. The color scale bar indicates gray value. e) Quantification of the LUC intensities in D. Values are means ± SD of 3 independent replicates. Tukey test was performed to detect the significant differences among different samples (*P* < 0.05), which were shown with letters. f) *β*-GAL activity assay indicated that NtBES1 repressed the expression of *NtPYL6a* and *NtPYL6b*. Values are means ± SD of 3 independent replicates. Two-tailed paired Student *t* test were performed to detect the significant differences (**, *P* < 0.01). g) ChIP assay revealed the enrichment of F1, F3, and F4 fragments present in the promoters of *NtPYL6a* and *NtPYL6b*. h) Y1H assay verified the binding effect of NtBES1 to the promoter fragments of *NtPYL6a* and *NtPYL6b*. i) EMSA (Electrophoretic mobility shift assay) confirmed that NtBES1 is bound to the F1 and F4 probes of *NtPYL6a* and *NtPYL6b* in vitro.

Our previous study shows that NtBES1 regulates the expression of important genes that respond to UV-B stress (Wang et al. 2024). Therefore, we examined the expression levels of *NtPYL6* in the existing *NtBES1-OE* and *ntbes1* plants to determine whether UV-B–induced expression of *NtPYL6* is dependent on the activity of NtBES1. The expression levels of *NtPYL6a* and *NtPYL6b* in *ntbes1* plants were separately 3.9- and 2.1-fold higher than that in WT plants, respectively, whereas their expression levels in *NtBES1-OE* plants were 69.8% and 53.9% significantly lower than that in WT plants (Fig. 2a), respectively. UV-B significantly induced the expression of *NtPYL6a* and *NtPYL6b* in WT plants by 6.3- and 3.7-fold (Fig. 2a), respectively. The expression levels of *NtPYL6a* and *NtPYL6b* in *ntbes1* plants treated with UV-B showed no significant differences from those in the UV-B–treated WT plants, but the expression levels of these 2 genes in *NtBES1-OE* plants treated with UV-B were 86.7% and 76.4% significantly lower than that in UV-B–treated WT plants (Fig. 2a), respectively. These results indicate that NtBES1 inhibits the expression of *NtPYL6* gene in tobacco. To further reveal the regulatory mechanism of NtBES1 on *NtPYL6* expression, the *35S:NtBES1-GR* plants were used to verify whether *NtPYL6* genes are direct targets of NtBES1. The GR tags will anchor the NtBES1-GR proteins to the membrane to inhibit their activities, but DEX could activate the fusion proteins by releasing them into the nucleus (Wang et al. 2021a). As shown in Fig. 2b, activation of the NtBES1-GR proteins by DEX treatment significantly repressed the expression levels of *NtPYL6a* and *NtPYL6b* by 44.5% and 30.2%, respectively, compared with that in the mock treatment. Moreover, DEX treatment still significantly inhibited the expression of *NtPYL6a* and *NtPYL6b* genes in the presence of a protein synthesis inhibitor CYC, suggesting that the inhibition of NtBES1 on *NtPYL6* expression requires no synthesis of new proteins, and therefore *NtPYL6* might be the direct target of NtBES1. Approximately 1.5-kb fragments upstream of the ATG were predicted to be the promoters of *NtPYL6a* and *NtPYL6b* genes by the Promoter-2.0 website. We then analyzed the putative cis-acting elements present in the promoter regions and found 7 and 3 E-box (CANNTG) elements in the promoters of *NtPYL6a* and *NtPYL6b* (Fig. 2c), respectively. BES1 has been shown to recognize and bind to the E-boxes to regulate the expression of its target genes (Li et al. 2009; Yu et al. 2011; Liang et al. 2020). To identify the essential cis-elements for the inhibition of NtBES1 on *NtPYL6* expression, various promoter fragments containing different E-box of *NtPYL6a* and *NtPYL6b* genes were used to drive the expression of *LUC* gene (Fig. 2c). Transient coexpression in tobacco leaves showed that NtBES1 significantly reduced the LUC intensities of *Pa1:LUC*, *Pa2:LUC*, and *Pa3:LUC* by 59.5%, 62.1%, and 90.1% (Fig. 2d, 2e), respectively. The LUC intensities of *Pb1:LUC* and *Pb2:LUC* co-injected with NtBES1 were also 76.7% and 79.3% significantly lower than that of control, respectively (Fig. 2d, 2e). *Pa1* and *Pb1* fragments were then fused to *β-Galactosidase* (*β-GAL*) as the reporter vectors. The *β*-GAL activity of the yeast strains containing *Pa1* reporter vectors and empty effector plasmid was 1.53 mU/mL, while *Pa1* reporter vectors co-transformed with NtBES1 produced 0.57 mU/mL *β*-GAL activities, which was 62.4% lower than that of control (Fig. 2f). Similarly, coexpression with NtBES1 also significantly reduced the *β*-GAL activity of *Pb1* reporter vectors (Fig. 2f).

ChIP-qPCR analysis showed that the F1 and F3 fragments of *NtPYL6a* were significantly enriched by 6.6- and 1.6-fold, respectively, and the F4 fragment of *NtPYL6b* was also 2.5-fold enriched (Fig. 2g). The F1 and F4 fragments were then used as bait sequences to perform Y1H assay, which further showed that NtBES1 can bind to the F1 and F4 fragments in yeast strains (Fig. 2h). NtBES1-HIS fusion proteins were purified and used to perform EMSA with the biotin-labeled F1 and F4 probes. Incubation of NtBES1-HIS with labeled F1 or F4 probes both gave rise to strong shift bands, and adding excessive unlabeled cold probes weakened or even eliminated the shift bands (Fig. 2i). Taken together, these results indicate that NtBES1 directly binds to the promoters of *NtPYL6a* and *NtPYL6b* to repress their expression.

### NtPYL6 mediates the ABA-induced accumulation of flavonols to enhance plant resistance to UV-B

**Figure 3:**
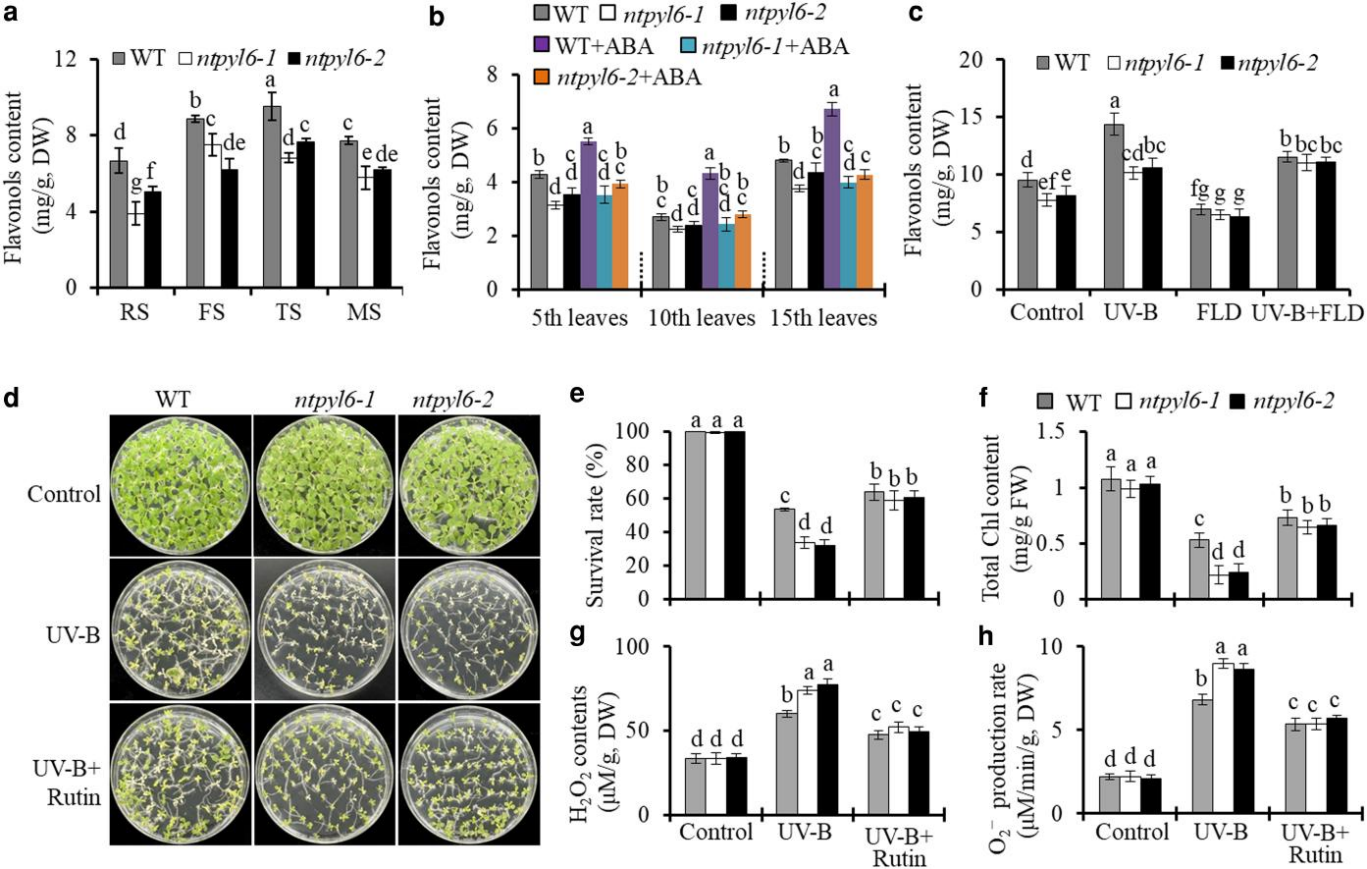
NtPYL6 mediates UV-B- and ABA-induced flavonol synthesis in tobacco. a) Flavonol content in the WT, *ntpyl6-1*, and *ntpyl6-2* leaves of different developmental stages. Abbreviations: FS, flowering stage; MS, maturation stage; RS, rosette stage; TS, topping stage. Values are means ± SD of 5 independent replicates. Tukey test was performed to detect the significant differences among different samples (*P* < 0.05), which were shown with letters. b) The flavonol content in different leaves of WT, *ntpyl6-1*, and *ntpyl6-2* plants treated with or without ABA. Tobacco plants, about 50 DAT were sprayed with 0.1 μM ABA every 4 h for 3 d. Leaves from the same position of 3 independent plants were mixed as a replicate. Values are means ± SD of 3 independent replicates. Tukey test was performed to detect the significant differences among different samples (*P* < 0.05), which were shown with letters. c) The flavonol content in the WT, *ntpyl6-1*, and *ntpyl6-2* plants treated with UV-B, FLD (fluridone), or UV-B + FLD. Values are means of 5 independent replicates ± SD. Tukey test was performed to detect the significant differences among different samples (*P* < 0.05), which were shown with letters. d) Phenotypes of WT, *ntpyl6-1*, and *ntpyl6-2* seedlings under control, UV-B, and UV-B + rutin treatment. e) Survival rates of the WT, *ntpyl6-1*, and *ntpyl6-2* seedlings under different treatments in D. f) Total Chl contents in the WT, *ntpyl6-1*, and *ntpyl6-2* seedlings under normal, UV-B, and UV-B + rutin treatment. g, h) The contents of H_2_O_2_ and O_2_^−^ in the WT, *ntpyl6-1*, and *ntpyl6-2* seedlings under different treatments. Values in E-H are means of 5 independent replicates ± SD. Tukey test was performed to detect the significant differences among different samples (*P* < 0.05), which were shown with letters.

An untargeted metabolomics detection was performed with the WT and *ntpyl6-1* leaves to provide clues for illustrating the enhanced sensitivity of *ntpyl6* mutant to UV-B. Leaves were collected from rosette stage, flowering stage, topping stage, and maturation stage, totaling 374 metabolites identified in all the leaf samples, among which 73 were flavonoids and 39 were phenolic acid (Fig. S2a-b, Table S1). Differentially accumulated metabolites (DAM) analysis revealed that compared with WT, the metabolic pathway with the greatest changes in *ntpyl6-1* leaves was the flavonols synthesis pathways (Fig. S2c). The flavonols contents in WT and *ntpyl6* plants were then confirmed by HPLC-UV method. The flavonol contents in the fresh leaves of *ntpyl6-1* from rosette stage, flowering stage, topping stage, and maturation stage were 41.4%, 15.2%, 28.4%, and 25.5% significantly lower than those in WT leaves (Fig. 3a), respectively. The flavonol contents in *ntpyl6-2* leaves were also 19.8% to 30.3% significantly lower than that in WT leaves from the 4 developmental stages (Fig. 3a). Moreover, the flavonol contents in the roots and flowers of *ntpyl6-1* and *ntpyl6-2* were also significantly lower than that in WT plants (Fig. S2d). Exogenous ABA treatment significantly increased the accumulation of flavonols in WT 5th, 10th, and 15th leaves by 28.8%, 60.2%, and 39.7% (Fig. 3b), respectively. However, the flavonol contents in these leaves of ABA-treated *ntpyl6-1* showed no significant differences from that of *ntpyl6-1* under normal conditions but were significantly lower than that of ABA-treated WT plants (Fig. 3b). Similarly, the flavonols contents in the 5th, 10th, and 15th leaves of *ntpyl6-2* treated with ABA were also significantly lower than that of ABA-treated WT plants but showed no significant differences from that of *ntpyl6-2* under normal conditions (Fig. 3b). These results indicate that NtPYL6 mediates the ABA-induced accumulation of flavonols in tobacco.

We have shown that UV-B radiation induced the accumulation of ABA in tobacco (Fig. 1f); therefore we proceeded to investigate whether NtPYL6 affects the biosynthesis of flavonols in response to UV-B. As expected, UV-B treatment significantly induced the flavonol accumulation by 51.1% in WT plants (Fig. 3c). The flavonol contents in UV-B–treated *ntpyl6-1* and *ntpyl6-2* were 30.2% and 27.6% significantly higher than that of control plants, respectively, and significantly lower than that of UV-B–treated WT plants, indicating that loss of function of NtPYL6 weakens the promotion effect of UV-B on flavonol synthesis. Next, fluridone (FLD), which is an effective inhibitor of ABA biosynthesis (Gamble and Mullet 1986), was used to further verify whether the induction of flavonol accumulation by UV-B depends on the synthesis of ABA. Compared with the control, FLD treatment significantly inhibited the biosynthesis of flavonols in WT, *ntpyl6-1*, and *ntpyl6-2* plants, but there were no significant differences among the flavonol contents of FLD-treated WT, *ntpyl6-1*, and *ntpyl6-2* plants (Fig. 3c). Moreover, the flavonol content in WT plants treated with UV-B + FLD was 19.7% significantly lower than that of UV-B–treated WT plants, indicating that inhibition of ABA synthesis repressed the flavonols accumulation induced by UV-B.

Next, we investigated whether exogenous flavonols can alleviate the sensitivity of *ntpyl6* mutants to UV-B. Rutins were used to perform the treatment, because they are important flavonols in tobacco, and exogenous rutin supplementation can improve plant resistance to abiotic stress (Chen et al. 2019). As described previously, UV-B radiation significantly reduced the survival rate, Chl content, and fresh and dry weight of WT plants, but these traits of WT plants treated with UV-B + rutin were significantly higher than that of UV-B–treated plants (Fig. 3d-f, S2e-f). Moreover, compared with UV-B treatment alone, the application of rutin significantly improved the survival rate, Chl content, and fresh and dry weight of *ntpyl6* plants, which did not significantly differ from that of WT plants treated with UV-B + rutin (Fig. 3d-f, S2e-f). Conversely, adding rutin to WT and *ntpyl6* plants significantly enhanced their antioxidant capacities and reduced the accumulation of ROS caused by UV-B, and the ROS levels did not significantly differ between WT and *ntpyl6* plants treated with UV-B + rutin (Fig. 3g-h, S2g-h). Collectively, these results indicate that UV-B induces ABA accumulation and *NtPYL6* expression in tobacco, and NtPYL6 mediates the ABA signal transduction to promote the accumulation of flavonols, thereby enhancing plant resistance to UV-B.

### ABA-induced flavonol accumulation depends on the activities of NtABFs in tobacco

**Figure 4:**
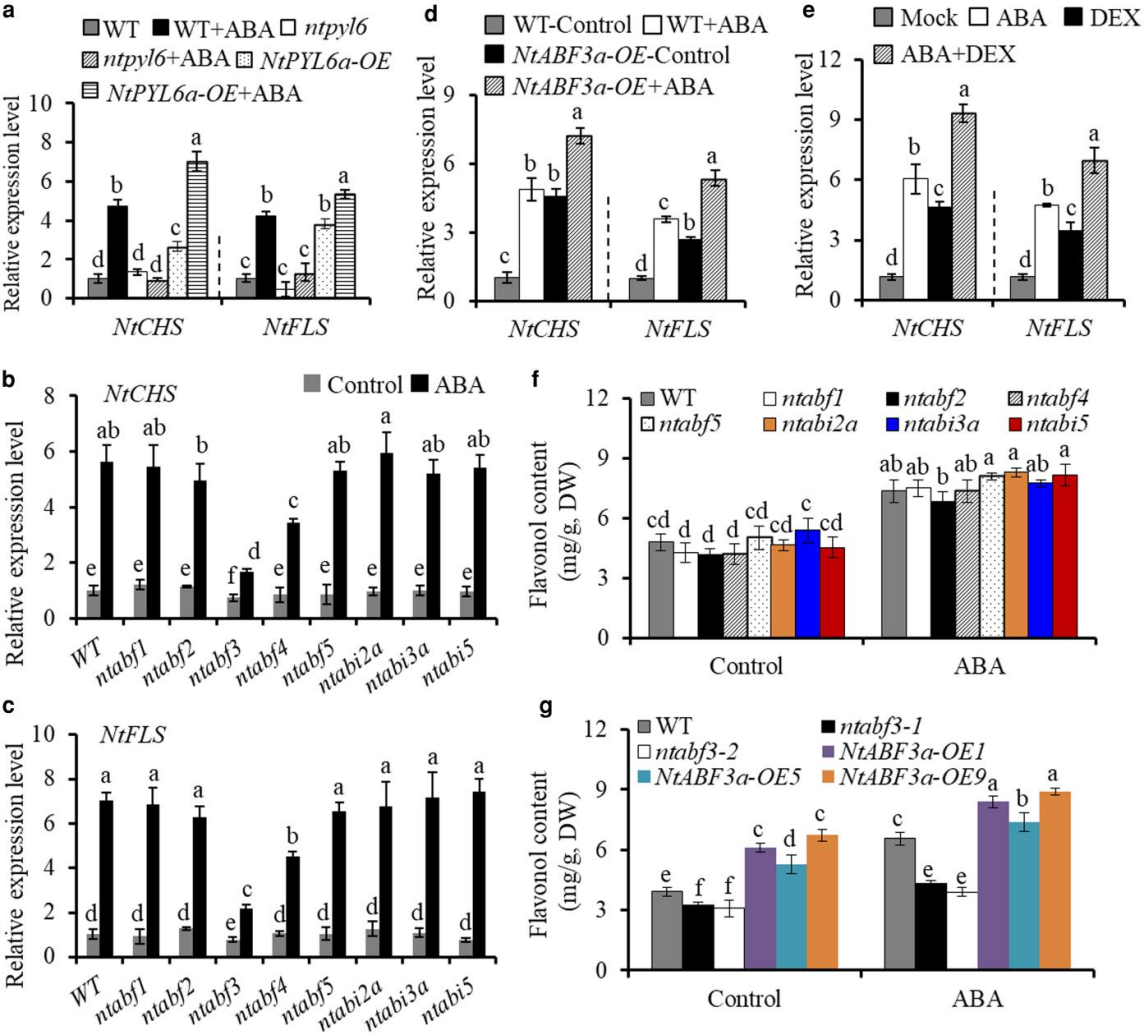
The induction of flavonols accumulation and related gene expression by ABA depend on the activity of NtABF3. a) Relative expression levels of *NtCHS* and *NtFLS* genes in different tobacco plants treated with or without ABA. Four-week-old WT, *ntpyl6*, and *NtPYL6a-OE* seedlings grown on the 1/2 MS medium were transferred to new mediums with or without 0.1 μM ABA for 1 h and then collected for gene expression analysis. b, c) Relative expression levels of *NtCHS* and *NtFLS* genes in different *ntabf* mutants treated with or without ABA. d) Relative expression levels of *NtCHS* and *NtFLS* in WT and *NtABF3a-OE* lines treated with or without ABA. e) Relative expression levels of *NtCHS* and *NtFLS* genes in *35S:NtABF3a-GR* seedlings treated with different chemicals. Four-week-old *35S:NtABF3a-GR* seedlings were transferred to the new mediums with 0.1 μM ABA, or 10 μM DEX, or ABA + DEX for 1 h. f) The flavonol content in the leaves of WT and different *ntabf* mutants treated with or without ABA. g) The flavonol content in the leaves of WT, *ntabf3*, and *NtABF3a-OE* plants treated with or without ABA. Four-week-old tobacco seedlings grown on the 1/2 MS medium were transferred to new mediums without or with 0.1 μM ABA for 3 d and then collected for flavonol measurement. Values are means of 3 independent replicates ± SD. Letters indicate significant differences detected via Tukey test (*P* < 0.05).

To reveal the regulatory mechanism of NtPYL6 in mediating ABA-induced flavonol accumulation, we detected the alteration of transcriptomes in the 15th leaves of WT and *ntpyl6-1* plants after ABA treatments. Exogenous ABA resulted in 5,216 and 3,764 differentially expressed genes (DEGs) in WT and *ntpyl6-1* plants, respectively, with 1,332 DEGs induced by ABA only in the WT plants (Fig. S3a-b, Table S2 and S3). Annotation of these 1,332 DEGs identified 6 genes that encode key enzymes involved in the phenylpropanoid pathway, including NtPAL (phenylalanine ammonia-lyase), Nt4CL (4-coumarate-coa ligase), NtCHS (chalcone synthase), NtFLS (flavonol synthase), NtLDOX (leucoanthocyanidin dioxygenase), and NtCCoAOMT1 (caffeoyl-coa o-methyl transferase 1) (Fig. S3c, Table S4). The expression of these 6 genes was significantly upregulated in WT plants by ABA but showed no significant differences in *ntpyl6-1* plants between control and ABA treatment (Fig. 4a, S3d, Table S4). In addition, overexpression of *NtPYL6a* and *NtPYL6a-GR* lines was generated to verify the regulation of NtPYL6 on the expression of these 6 genes (Fig. S3e). Overexpression of *NtPYL6a* significantly upregulated the expression levels of these 6 genes, and ABA treatment could further enhance the expression of *NtCHS*, *NtFLS*, *NtPAL*, *NtLDOX*, and *NtCCoAMT1* genes in the *NtPYL6a-OE* plants (Fig. 4a, S3d).

Moreover, activation of NtPYL6a-GR fusion proteins by DEX treatment significantly induced the expression of these 6 genes. The expression levels of *NtCHS* and *NtFLS* in *35S:NtPYL6a-GR* seedlings treated with ABA + DEX were significantly higher than those in seedlings treated with ABA or DEX alone (Fig. S3f and S4a). Taken together, these results indicate that NtPYL6 mediates the ABA-induced expression of flavonols synthesis related genes in tobacco.

PYL proteins binding with ABA could activate the ABF transcription factors through the core signaling pathways, thereby regulating the expression of various ABA-responsive genes (Kobayashi et al. 2005; Furihata et al. 2006; Zhao et al. 2016). To test whether NtABFs are involved in regulating the ABA-induced flavonols synthesis, a total of 18 putative ABF homologous proteins were identified from the *N. tabacum* genome (Table S5). They were clustered into 8 groups (Fig. S4b), and the members within each group shared high sequence identities, so conserved sgRNAs were generated to knock out all the genes in each group, giving rise to *ntabf1*, *ntabf2*, *ntabf3*, *ntabf4*, *ntabf5*, *ntabi2*, *ntabi3*, and *ntabi5* mutants (Fig. S4c and S4d), respectively. The expression levels of *NtCHS* and *NtFLS* in *ntabf3* were significantly lower than those in WT plants, but their transcription showed no significant differences between WT and other *ntabf* mutants. ABA treatment significantly induced the expression of *NtCHS* and *NtFLS* in WT plants, but their expression levels in *ntabf3* and *ntabf4* plants treated with ABA were significantly lower than those in WT plants treated with ABA (Fig. 4b and 4c). In contrast, the expression levels of *NtCHS* and *NtFLS* in *ntabf1*, *ntabf2*, *ntabf5*, *ntabi2a*, *ntabi3a*, and *ntabi5* plans treated with ABA showed no significant differences with those in WT plants treated with ABA (Fig. 4b and 4c). In addition, we found that the expressions of *NtPAL*, *Nt4CL*, *NtLDOX*, and *NtCCoAMT1* genes in the 8 *ntabf* mutants treated with or without ABA showed no significant differences with those in WT plants treated with or without ABA (Fig. S5), respectively. Overexpression of *NtABF3a* and *NtABF3a-GR* lines was generated to further verify its regulation on the expression of *NtCHS* and *NtFLS* genes (Fig. S4e). The expression levels of *NtCHS* and *NtFLS* in *NtABF3a-OE* lines were significantly higher than those in WT plants, and ABA treatment to the *NtABF3a-OE* plants could further promote the expression of these 2 genes, which were even higher than those in WT plants treated with ABA (Fig. 4d). Similarly, DEX treatment to *35S:NtABF3a-GR* lines significantly enhanced the expression of *NtCHS* and *NtFLS*, and their expression showed the highest levels in the *35S:NtABF3a-GR* plants treated with DEX + ABA (Fig. 4e). These results indicate that the induction of ABA on the expression of *NtCHS* and *NtFLS* genes depends on the activities of NtABF3.

To test whether the NtABFs were involved in ABA-induced flavonol synthesis, the contents of flavonols in different mutants were detected. As shown in Fig. 4f, the flavonol contents in the leaves of *ntabf1*, *ntabf2*, *ntabf4*, *ntabf5*, *ntabi2a*, *ntabi3a*, and *ntabi5* mutants did not significantly differ from that in the WT plants. ABA treatment to these mutants significantly increased their accumulation of flavonols, which also showed no significant differences with that in WT plants treated with ABA (Fig. 4f). However, the flavonol contents in *ntabf3-1* and *ntabf3-2* significantly reduced by 16.7% and 21.1% compared with that in WT plants, respectively. ABA treatment increased the flavonols contents in WT plants by 67.5%, but the flavonol contents in *ntabf3-1* and *ntabf3-2* mutants treated with ABA only increased by 33.0% and 26.3% (Fig. 4g), respectively. In addition, the flavonol contents in 3 independent *NtABF3a-OE* lines were all significantly higher than that in the WT plants, and ABA treatment to the *NtABF3a-OE* lines further enhanced the accumulation of flavonols (Fig. 4g). These results indicate that NtABF3 was involved in mediating ABA-induced flavonols biosynthesis in tobacco.

### NtABF3 directly binds to the promoters of *NtCHS* and *NtFLS* to induce their expression and flavonol accumulation

**Figure 5:**
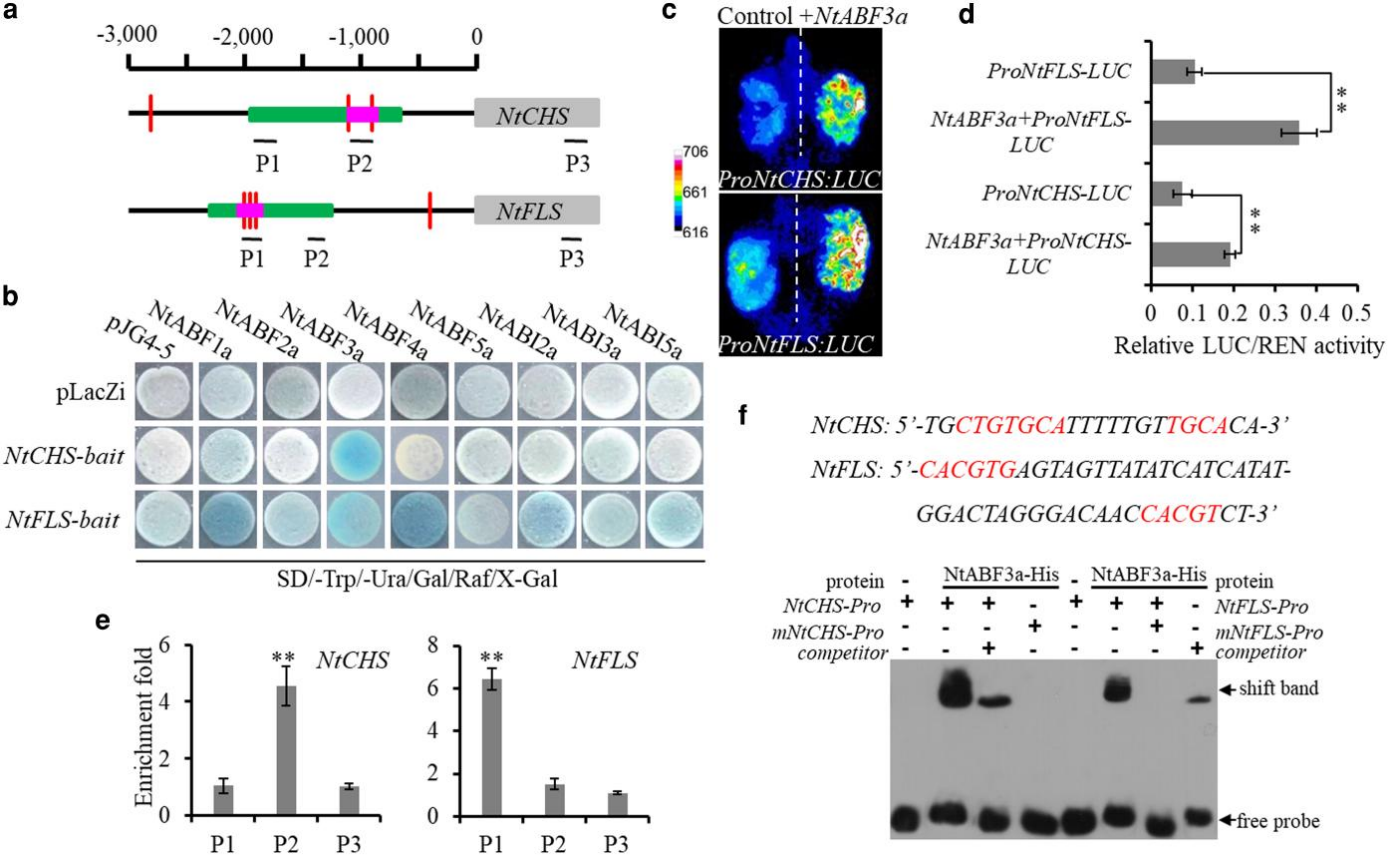
NtABF3a directly targetes *NtCHS* and *NtFLS* to induce their expression. a) Distribution of putative ABREs (ABA-responsive element) present in the upstream 3-kb sequences of *NtCHS* and *NtFLS* genes. Red lines represent the putative ABREs. Purple boxes indicate the fragments used for Y1H assay. Green boxes indicate the fragments used for dual-LUC assay. P1, P2, and P3 indicate the fragments detected in the subsequent ChIP assay. b) Y1H assay showed that NtABF3a, rather than other NtABFs, bound to the ABRE fragments of both *NtCHS* and *NtFLS* genes. c) LUC fluorescence of *pNtCHS-LUC* and *pNtFLS-LUC* plasmids co-injected with *NtABF3a* or empty vector. The color scale bar indicates gray value. d) Quantification of the LUC intensities in C. e) Enrichment folds of different *NtCHS* and *NtFLS* fragments in ChIP assay. Values are means of 3 independent replicates ± SD. Asterisks indicate the significant differences, detected via 2-tailed paired Student *t* test (**, *P* < 0.01). f) EMSA verified the in vitro binding of NtABF3a to the ABREs present in the *NtCHS* and *NtFLS* promoters. Red letters indicate the putative ABREs, and the ACGT in the ABREs were replaced with AAAT to generate mutant probes. Un-labeled probes were used as competitors.

To investigate whether the 6 flavonols related DEGs are targets of NtABFs, their 3-kb putative promoter sequences were analyzed to identify the ABRE (ABA-responsive element, ACGTGG/TC) cis-elements. There were 3, 4, 2, 4, 3, and 3 ABREs presented in the promoters of *NtCHS*, *NtFLS*, *NtPAL*, *Nt4CL*, *NtLDOX*, and *NtCCoAMT1*, respectively (Fig. 5a and S6a). Promoter fragments of each gene containing the ABRE elements and 1 *NtABF* gene from each group were cloned to determine their regulatory effect on the 6 DEGs. Y1H assay showed that NtABF3a could bind to both the promoter fragments of *NtCHS* and *NtFLS* containing the ABREs, while the other 7 NtABFs showed no simultaneous binding effects to the probes of these 2 genes (Fig. 5b). Consistently, we found that NtABF3a was able to significantly enhance the LUC activities driven by the promoters of *NtCHS* and *NtFLS* genes (Fig. 5c and 5d), while the other 7 NtABFs showed no promotion effect on these 2 LUC reporter vectors (Fig. S6b). However, all the NtABFs examined showed no effect on *NtPAL*, *Nt4CL*, *NtLDOX*, and *NtCCoAMT1* genes in the Y1H and dual-LUC assays (Fig. S6b and S6c). ChIP-qPCR and EMSA were then performed to verify the binding of NtABF3a to promoter fragments of *NtCHS* and *NtFLS*. After immunoprecipitation, the P2 promoter fragment of *NtCHS* and the P1 fragment of *NtFLS* were significantly enriched (Fig. 5e). The NtABF3a-HIS fusion protein could bind to the labeled *NtCHS* and *NtFLS* probes containing ABREs, while the binding effect decreased when unlabeled probes were added. Mutation of the ABREs (replacing ACGT with AAAT) in the labeled *NtCHS* and *NtFLS* probes completely abolished the binding ability of NtABF3a-HIS (Fig. 5f). Moreover, EMSA also showed that NtABF2 might bind to the *NtCHS* probes, while NtABF4, NtABI5, and NtABF1 might target *NtFLS* probes (Fig. S6d and S6e). Collectively, these results indicate that NtABF3a directly binds to the promoters of *NtCHS* and *NtFLS* genes to induce their expression levels.

**Figure 6:**
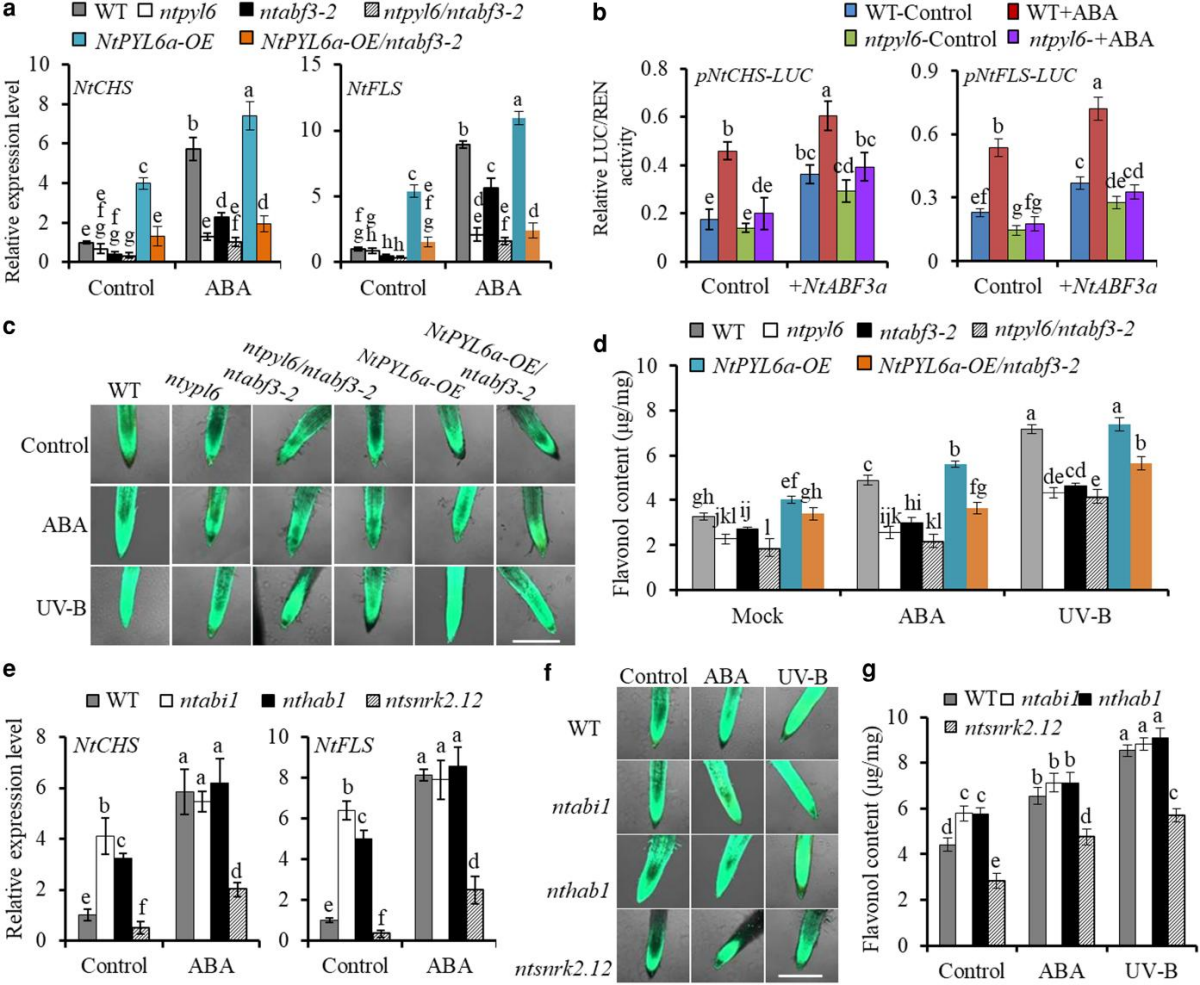
Activities of the core ABA signal pathway members affect flavonol accumulation in tobacco. a) Relative expression levels of *NtCHS* and *NtFLS* genes in WT, *ntpyl6*, *ntabf3-2*, *ntpyl6/ntabf3-2*, *NtPYL6a-OE*, and *NtPYL6a-OE/ntabf3-2* plants treated with or without ABA. Four-week-old tobacco seedlings grown on 1/2 MS medium were transferred to new mediums without or with 0.1 μM ABA for 1 h and then collected for gene expression analysis. b) LUC intensities of *pNtCHS-LUC* and *pNtFLS-LUC* co-injected with *NtABF3a* or not into the protoplasts of WT and *ntpyl6* treated with or without ABA. c) DPBA staining of the flavonols in the roots of WT, *ntpyl6*, *ntabf3-2*, *ntpyl6/ntabf3-2*, *NtPYL6a-OE*, and *NtPYL6a-OE/ntabf3-2* plants. The bar represents 50 µm and applies to all subpanels. d) Flavonol content in the seedlings of WT, *ntpyl6*, *ntabf3-2*, *ntpyl6/ntabf3-2*, *NtPYL6a-OE*, and *NtPYL6a-OE/ntabf3-2* plants. Four-week-old tobacco seedlings grown on the 1/2 MS medium were transferred to new mediums, which were treated with 0.1 μM ABA for 3 d or UV-B for 30 min. The UV-B–treated seedlings were grown under normal conditions for 3 d after treatment, and then all the seedlings were collected for flavonol measurement. e) Relative expression levels of *NtCHS* and *NtFLS* genes in WT, *ntabi1*, *nthab1*, and *ntsnrk2.12* plants treated with or without ABA. Four-week-old tobacco seedlings grown on 1/2 MS medium were transferred to new mediums without or with 0.1 μM ABA for 1 h and then collected for gene expression analysis. f) DPBA staining of the flavonols in the roots of WT, *ntabi1*, *nthab1*, and *ntsnrk2.12* plants. The bar represents 50 μm and applies to all subpanels. G, Flavonols contents in the WT, *ntabi1*, *nthab1*, and *ntsnrk2.12* plants. Values are means of 3 independent replicates ± SD. Letters indicate significant differences detected via Tukey's test (*P* < 0.05).

To verify whether NtPYL6 and NtABF3 work in the same way to mediate ABA-induced flavonols biosynthesis in tobacco, *ntpyl6/ntabf3-2* double mutant and *NtPYL6a-OE/ntabf3-2* lines were generated by crossing. The expression levels of *NtCHS* and *NtFLS* in *ntpyl6/ntabf3-2* double mutant were significantly lower than those in WT plants but showed no significant differences from those in the *ntabf3-2* plants (Fig. 6a and 6b). After ABA treatment, the expression levels of *NtCHS* and *NtFLS* in *ntpyl6*, *ntabf3-2*, and *ntpyl6/ntabf3-2* mutants were all significantly lower than those in WT plants. The expression of *NtCHS* and *NtFLS* in *ntpyl6/ntabf3-2* double mutants treated with ABA was significantly lower than that in *ntabf3-2* treated with ABA but showed no significant differences from those in *ntpyl6* treated with ABA (Fig. 6a). Moreover, we found that overexpression of *NtPYL6a* significantly enhanced the expression of *NtCHS* and *NtFLS*, but their expression levels in *NtPYL6a-OE/ntabf3-2* plants showed no significant differences from those in WT plants. ABA treatment further induced the expression of *NtCHS* and *NtFLS* in *NtPYL6a-OE* lines, but this induction was almost abolished in *NtPYL6a-OE/ntabf3-2* plants (Fig. 6a), indicating that the induction of ABA-NtPYL6 on the expression of these 2 genes depends on the activity of NtABF3. The promoter of *NtCHS* and *NtFLS* was then used to drive the expression of *LUC* gene in the WT or *ntpyl6* protoplasts with or without *NtABF3a*. When the *pNtCHS-LUC* or *pNtFLS-LUC* plasmids were transformed into the protoplasts alone, ABA treatment significantly enhanced the LUC intensity in the WT protoplasts, but the LUC intensities showed no significant differences between the ABA-treated and untreated *ntpyl6* protoplasts (Fig. 6b). Co-transformed with *NtABF3a* significantly increased the LUC intensities of *pNtCHS-LUC* and *pNtFLS-LUC* in both the WT and *ntpyl6* protoplasts, and ABA treatment further enhanced this promoting effect of NtABF3a on the LUC intensities of the 2 plasmids in the WT protoplasts. However, ABA treatment in the *ntpyl6* protoplasts failed to promote the LUC intensities of *pNtCHS-LUC* and *pNtFLS-LUC* co-transformed with *NtABF3a* (Fig. 6b). These results indicate that the activation of NtABF3 by ABA might depend on the activity of NtPYL6.

DPBA staining showed that the flavonols fluorescence in the roots of *ntpyl6*, *ntabf3-2*, and *ntpyl6/ntabf3-2* was weaker than that in the WT roots. ABA treatment enhanced the fluorescence in the roots of WT plants, but that in the *ntpyl6*, *ntabf3-2*, and *ntpyl6/ntabf3-2* roots was less affected (Fig. 6c). There was strong fluorescence signal in the roots of *NtPYL6a-OE* plants, and ABA treatment further increased the fluorescence in these *NtPYL6a-OE* lines. However, the fluorescence in the roots of *NtPYL6a-OE/ntabf3-2* plants was weak, and after ABA treatment their fluorescence was obviously less than that in *NtPYL6a-OE* roots treated with ABA (Fig. 6c). HPLC analysis confirmed that the flavonol contents in the *ntpyl6*, *ntabf3-2*, and *ntpyl6/ntabf3-2* seedlings were significantly lower than that in the WT. ABA treatment significantly induced flavonols accumulation in the *ntabf3-2* seedlings, which was still significantly lower than that in the WT treated with ABA (Fig. 6d). The flavonol contents in the *ntpyl6/ntabf3-2* seedlings treated with or without ABA were significantly lower than that in the *ntabf3-2* but showed no significant differences with that in the *ntpyl6* treated with or without ABA (Fig. 6d), respectively. The content of flavonols in *NtPYL6a-OE* was significantly higher than that in the WT, but the content in *NtPYL6a-OE/ntabf3-2* showed no significant difference from that in WT. Moreover, ABA treatment significantly increased the flavonols content of *NtPYL6a-OE* seedlings, and the content in *NtPYL6a-OE/ntabf3-2* treated with ABA was significantly lower than that in *NtPYL6a-OE* treated with ABA (Fig. 6d). Collectively, these results indicate that the promotion of flavonols accumulation and related gene expression by ABA-NtPYL6 depends on the activities of NtABF3.

### The ABA-NtPYL6 signaling pathway enhances flavonols biosynthesis and plant resistance to UV-B

ABA has been shown to activate the ABF transcription factors through the core ABA-PYL-PP2C-SnRK signaling pathway (Fujii et al. 2009; Park et al. 2009; Zhu 2016). Next, we identified the key PP2C and SnRK members involved in ABA-NtPYL6-NtABF3–induced flavonol biosynthesis in tobacco. A total of 20 putative PP2C members were identified from the *N. tabacum* genome, and they were clustered into 10 pairs (Fig. S7a, Table S5). RT-qPCR analysis showed that *NtHAB1*, *NtABI1*, *NtHAB2*, and *NtHAI3* genes had high expression levels in tobacco leaves of different developmental stages (Fig. S7b). One member of each pair was cloned and used to detect their interaction with NtPYL6a. LUC assay indicated strong interaction between NtABI1a and NtPYL6a, as well as between NtHAB1a and NtPYL6a (Fig. S7c). Similarly, a total of 31 putative SnRK2 members were identified and named as NtSnRK2.1-2.13 based on their evolutionary relationship (Fig. S8a, Table S5). *NtSnRK2.1*, *NtSnRK2.2*, *NtSnRK2.9*, and *NtSnRK2.12* showed high expression levels in tobacco leaves (Fig. S8b); thus we detected their interaction with NtABI1 and NtHAB1. NtSnRK2.12a showed strong interaction with both NtABI1a and NtHAB1a (Fig. S8c). Y2H further verified the interaction between NtPYL6a and NtABI1a/NtHAB1a, as well as between NtABI1a/NtHAB1a and NtSnRKY2.12 (Fig. S9a, S9b). Phosphorylation assays showed that coexpression of NtSnRKY2.12-myc and NtABF3a-flag in the leaves of *N. benthamiana* greatly increased the phosphorylation of NtABF3a-flag proteins, and exogenous ABA application or UV-B treatment can further enhance the phosphorylation of NtABF3a-flag proteins by NtSnRKY2.12-myc (Fig. S9c, S9d). These results indicate that NtABI1, NtHAB1, and NtSnRK2.12 might be the key PP2C or SnRK members involved in NtPYL6 mediated ABA core signaling pathway.

To test whether *NtABI1*, *NtHAB1*, and *NtSnRK2.12* were involved in ABA induced flavonols biosynthesis, their loss-of-function mutants were generated by Crispr/cas9 (Fig. S10). The expression levels of *NtCHS* and *NtFLS* in *ntabi1* and *nthab1* mutants were significantly higher than those in WT plants, while their expression levels in *ntsnrk2.12* were significantly lower than those in WT plants (Fig. 6e). After ABA treatment, the expression of *NtCHS* and *NtFLS* in *ntabi1* and *nthab1* mutants showed no significant differences with those in WT plants, but their expression levels in *ntsnrk2.12* were significantly lower than those in WT plants (Fig. 6e). Consistent with the gene expression pattern, the flavonols contents in *ntabi1* and *nthab1* roots were significantly higher than that in WT roots, and the *ntsnrk2.12* roots contained significantly less flavonols than WT plants (Fig. 6f, 6g). After ABA treatment, the flavonols contents in *ntabi1* and *nthab1* mutants showed no significant differences with those in WT plants, but the content in *ntsnrk2.12* was significantly lower than those in WT plants (Fig. 6f, 6g). These results indicate that NtABI1, NtHAB1, and NtSnRK2.12 were involved in ABA-induced flavonols biosynthesis. *pNtCHS-LUC* and *pNtFLS-LUC* plasmids were separately transformed into the protoplasts of different mutants alone or with *NtABF3a*. When transformed alone, the LUC intensities of *pNtCHS-LUC* or *pNtFLS-LUC* in *ntabi1* and *nthab1* protoplasts were significantly higher than those in the WT protoplasts, but their intensities in *ntsnrk2.12* protoplasts were significantly lower than those in WT protoplasts (Fig. S9e). Co-transformed with *NtABF3a* significantly increased the LUC intensities of *pNtCHS-LUC* and *pNtFLS-LUC* in *ntabi1* and *nthab1* protoplasts, which were even higher than those in WT protoplasts co-transformed with *NtABF3a*. ABA treatment further enhanced this promoting effect of NtABF3a on the LUC intensities of the 2 plasmids in the WT protoplasts, but the LUC intensities of *pNtCHS-LUC* + *NtABF3a* and *pNtFLS-LUC* + NtABF3a in *ntabi1* or *nthab1* protoplasts showed no significant changes between control and ABA treatment (Fig. S9e). Moreover, the LUC intensities of *pNtCHS-LUC* + *NtABF3a* and *pNtFLS-LUC* + NtABF3a in *ntsnrk2.12* protoplasts treated with or without ABA were significantly lower than those in WT protoplasts treated with or without ABA (Fig. S9e), respectively. Collectively, these results indicate that NtPYL6-NtABI1/NtHAB1-NtSnRK2.12 might constitute the core ABA signaling pathway to regulate ABA induced NtABF3 activities and flavonols accumulation in tobacco.

**Figure 7:**
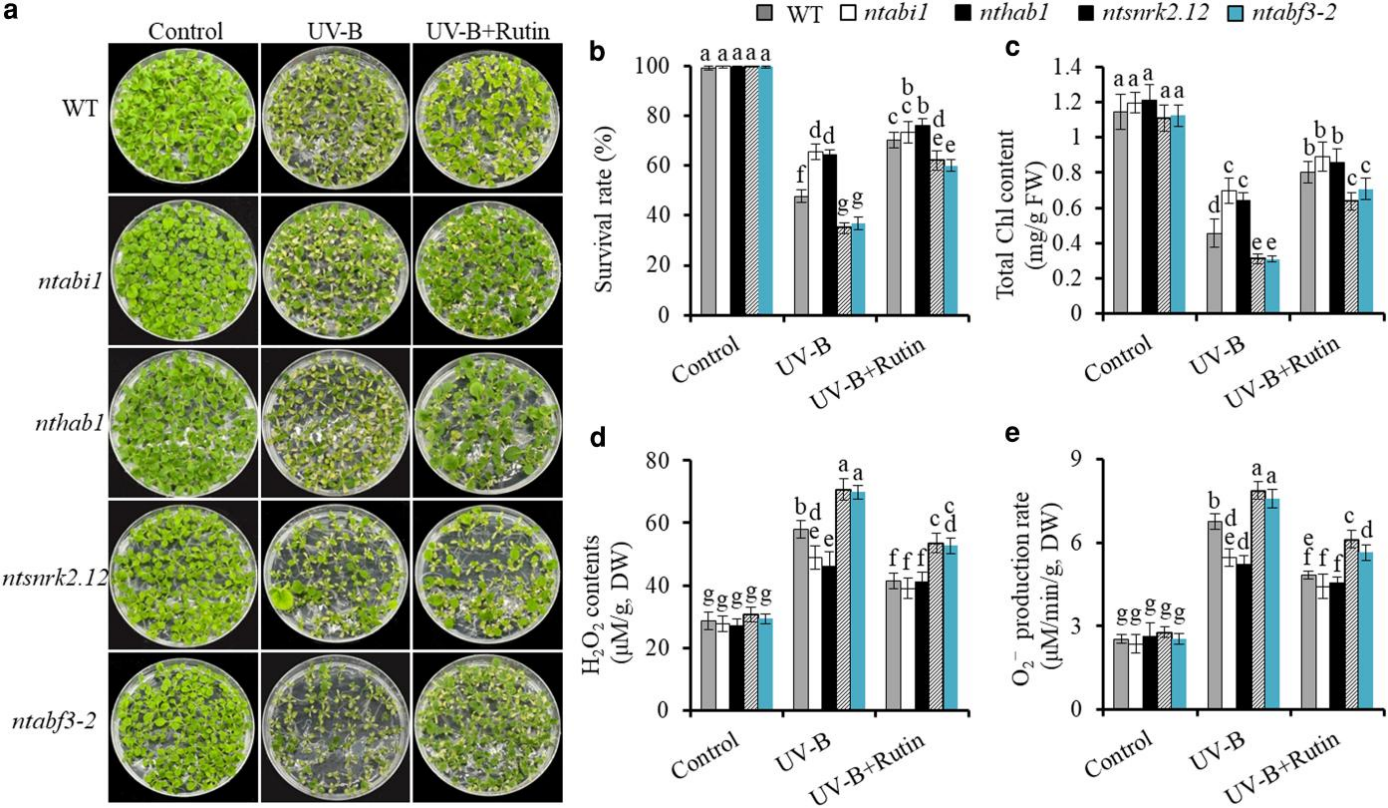
The core ABA signal pathway members are involved in plant resistance to UV-B. **a)** Phenotypes of WT, *ntabi1*, *nthab1*, *ntsnrk2.12*, and *ntabf3-2* seedlings under different treatment. **b)** Survival rates of the WT, *ntabi1*, *nthab1*, *ntsnrk2.12*, and *ntabf3-2* seedlings under different treatment. **c)** Total Chl contents in the WT, *ntabi1*, *nthab1*, *ntsnrk2.12*, and *ntabf3-2* seedlings under different treatments. **d, e)** The contents of H_2_O_2_ and O_2_^−^ in the WT, *ntabi1*, *nthab1*, *ntsnrk2.12*, and *ntabf3-2* seedlings under different treatments. Values are means ± SD of 5 independent replicates. Letters indicate significant differences detected via Tukey test (*P* < 0.05).

Next, we investigated whether these ABA signaling members were involved in plant resistance to UV-B. The WT, *ntabi1*, *nthab1*, *ntsnrk2.12*, and *ntabf3-2* seedlings showed no obvious differences under normal conditions. However, *ntabi1* and *nthab1* seedlings treated with UV-B exhibited significantly higher survival rates than UV-B–treated WT plants, whereas the survival rates of *ntsnrk2.12* and *ntabf3-2* were significantly lower than that of WT after UV-B treatment (Fig. 7a, 7b). Consistently, the Chl contents and the fresh and dry weight of *ntabi1* and *nthab1* leaves treated with UV-B were significantly higher than that of UV-B–treated WT leaves, while these traits of *ntsnrk2.12* and *ntabf3-2* plants treated with UV-B were significantly lower than WT (Fig. 7c, S11a-b). In contrast, after UV-B treatment, the accumulation of H_2_O_2_ and O_2_^−^ significantly decreased in *ntabi1* and *nthab1* seedlings compared with that of WT, whereas the contents of H_2_O_2_ and O_2_^−^ in *ntsnrk2.12* and *ntabf3-2* were significantly higher than that of WT (Fig. 7d, 7e). Moreover, we found that compared with UV-B treatment alone, adding rutin to WT, *ntabi1*, *nthab1*, *ntsnrk2.12*, and *ntabf3-2* seedlings significantly improved their survival rates, Chl contents, and antioxidant capacities but reduced their H_2_O_2_ and O_2_^−^ accumulation under UV-B treatment (Fig. 7a-d, S11c-d). Additionally, we observed that *NtPYL6a-OE* seedlings exhibited stronger UV-B resistance than the WT plants (Fig. S12). Collectively, these results indicate that members of the core ABA-PYL-PP2C-SnRK signaling pathway were involved in regulating flavonols biosynthesis and plant resistance to UV-B in tobacco.

## Discussion

ABA could enhance the accumulation of flavonols in plants (Watkins et al. 2017; Gai et al. 2020), which has been suggested as an ancestral trait of land plants shielding against UV-B radiation (Rozema et al. 1997; Brunetti et al. 2019). However, the mechanism by which ABA signaling contributes to flavonols biosynthesis and plant resistance to UV-B radiation still needs to be further clarified. Here, we showed that UV-B enhanced ABA accumulation and the expression of *NtPYL6* gene in tobacco and further proved that NtBES1 directly binds to the promoter of *NtPYL6* to mediate the induction of its transcription by UV-B. The enhanced ABA-NtPYL6 signaling pathway activates the NtABF3, which directly induced the expression of *NtCHS* and *NtFLS* genes to promote the synthesis of flavonols, thereby enhancing tobacco resistance to UV-B. Our results reveal the detailed regulatory mechanism of NtPYL6-mediated ABA signal in enhancing plant UV-B resistance by inducing flavonol accumulation.

BES1 has been shown to participate in regulating plant resistance to various environmental stresses, including UV-B radiation (Liang et al. 2018; Nolan et al. 2020; Yao et al. 2022). When plants suffer UV-B, the interaction between UVR8 and BES1 will be enhanced thereby initiating the BES1-mediated tradeoff between plant growth and UV-B defense responses (Liang et al. 2019; Liang et al. 2020). Our analysis also confirmed that NtUVR8 can remove the inhibition of NtBES1 on *NtPYL6a* expression under UV-B treatment (Fig. S13a-b). Therefore, the inhibition of BES1 by UVR8 has different strategies to induce flavonols accumulation in response to UV-B. One is enhancing the activities of flavonols synthesis promoting factors MYB11/12/111 (Stracke et al. 2007; Liang et al. 2018, 2020), and the other one is to inhibit the activities of flavonol synthesis inhibiting factor MYB27 (Wang et al. 2024). Here, we revealed a way through which BES1 can regulate the synthesis of flavonols and plant response to UV-B. We showed that NtBES1 directly bound to the promoter of *NtPYL6* to repress its expression, and UV-B removed this inhibition to enhance the ABA signaling (Fig. 2). The enhanced ABA-NtPYL6 signaling pathway activated NtABF3, which further directly induced the expression of *NtCHS* and *NtFLS*, leading to elevate the accumulation of flavonols in tobacco (Figs. 4–6). Moreover, ABA has been well-characterized to coordinate with other plant hormones to modulate the balance of plant growth and abiotic stress responses (Claeys and Inzé 2013; Julkowska and Testerink 2015; Liu et al. 2022). Therefore, BES1 might serve as a key coordinator of ABA signal and UV-B responses in plants. However, more efforts are still needed to elucidate the roles of BES1 in regulating the biosynthesis of ABA and the activities of other members in the core ABA signaling pathway in response to UV-B.

The ABFs are important regulators of numerous ABA-responsive genes in ABA signaling pathway (Fujii et al. 2009; Zhu 2016), and they also act as critical connectors between the ABA signaling pathway and other signaling pathways (Bhagat et al. 2021; Song et al. 2022; Xian et al. 2024). However, the roles of different ABF members might be differentiated in mediating the crosstalk of ABA and other signaling pathways. For instance, the DELLA protein RGL2 (RGA-LIKE 2) forms a heterodimer by specifically interacting with ABI4 to mediate the antagonism between ABA and GA (Gibberellins) (Xian et al. 2024). ABI5 has been shown to physically interact with HY5 to fine-tune light and ABA signaling in Arabidopsis (Bhagat et al. 2021). Osmotic stress dramatically induced the expression of *StABF2/StAREB2*, *StABF3/StAREB4*, and S*tABF4/StAREB1* in *Solanum tuberosum* (Yoshida et al. 2015), and ABA acted through ABF3 and ABF4 to accelerate flowering in Arabidopsis under drought stress (Hwang et al. 2019). We showed here NtABF3 was involved in regulating flavonols accumulation and plant resistance to UV-B radiation (Figs. 6c-d, 7), suggesting that NtABF3 might be a connector linking ABA and UV-B signaling pathway. When suffering UV-B, ABF3 might be predominantly activated by enhanced ABA signal to promote the synthesis of flavonols, so it will be interesting to explore the full roles of ABF3 in regulating plant resistance to UV-B.

ABA has been suggested to enhance the interaction between ABI5 and HY5, which might further activate MYB12 and MYB111, thereby promoting the expression of flavonol synthesis–related genes and the accumulation of flavonols in Arabidopsis (Stracke et al. 2010; Brunetti et al. 2019; Bhagat et al. 2021). We showed here that NtABF3 is directly bound to the ABRE cis-elements present in the promoters of *NtCHS* and *NtFLS* to induce their expression (Fig. 5). We then tested the interaction of NtABF3 and NtHY5 but found that there was no interaction between these 2 proteins (Fig. S13c-d). Therefore, the regulation of NtABF3 on flavonol accumulation might differ from that of AtABI5, and our results revealed a way in which ABA promotes the biosynthesis of flavonols. Our EMSA analysis indicated that there was binding effect of NtABF1, NtABF4, and NtABI5 to the *NtFLS* probes, and NtABF2 could bind to the *NtCHS* probes containing ABRE elements (Fig. S6d and S6e). Moreover, ABA treatment could still induce the expression of these 2 genes and the accumulation of flavonols in *ntabf3-1* and *ntabf3-2* mutants (Fig. 4b-c), suggesting that in addition to NtABF3, other NtABF transcription factors might also be involved in regulating ABA induced flavonols biosynthesis. Several other transcription factors, including NtMYB12 and NtWRKY11b, have also been shown to promote flavonols biosynthesis in tobacco by directly targeting the structural genes in this pathway (Wang et al. 2021a, 2021b). However, whether these regulators respond to ABA signaling and interact with NtABFs to jointly regulate flavonols synthesis and related gene expression still needs to be further clarified.

We suggested here that NtPYL6-NtABI1/NtHAB-NtSnRK2.12 might constitute the core ABA signaling pathways to induce flavonols synthesis in response to UV-B (Figs. 6f-g, 7). However, we found that ABA treatment could still significantly promote the accumulation of flavonols in *ntabi1*, *nthab1*, and *ntsnrk2.12* mutants (Fig. 6f-g), suggesting that the induction of ABA on flavonols synthesis might be independent of the core signaling pathway. This inconsistency might be mainly caused by 2 reasons. One is the functional redundancy of homologous proteins. There were 20 putative *PP2C* and 31 *SnRK2* genes in the genome of allotetraploid tobacco (Fig. S7, S8). We could not rule out the possibility that there might be other NtPYL6-NtPP2C-NtSnRK2 signaling pathway to mediate the ABA-induced flavonol biosynthesis. Another possibility is that the regulation of flavonol biosynthesis by ABA-NtPYL6 might not be entirely achieved through the core ABA signaling pathway. AtPYL8 interacts with AtMYB77, AtMYB44, and AtMYB73 to enhance auxin signaling to promote the growth of lateral roots in Arabidopsis, and this regulation is independent of the core ABA signaling pathway (Zhao et al. 2014). In the presence of ABA, AtPYL6 interacts with MYC2 to regulate the expression pattern of *JAZ6* (*JASMONATE ZIM-DOMAIN 6*) and *JAZ8* genes, indicating that there is a putative link between ABA and JA signaling independently of the core ABA signaling pathway (Aleman et al. 2016). We also found that the expression levels of several *NtJAZ* genes were differentially regulated by ABA in WT and *ntpyl6* mutants (Tables S2 and S3). JAZ proteins have been shown to inhibit the biosynthesis of anthocyanins in Arabidopsis (Qi et al. 2011). Therefore, it will be interesting to further explore whether ABA-NtPYL6 affects the abundance and activities of JAZ proteins independently of the core ABA signaling pathway, and whether ABA-NtPYL6-JAZ is involved in regulating the biosynthesis of flavonols in tobacco.

**Figure 8:**
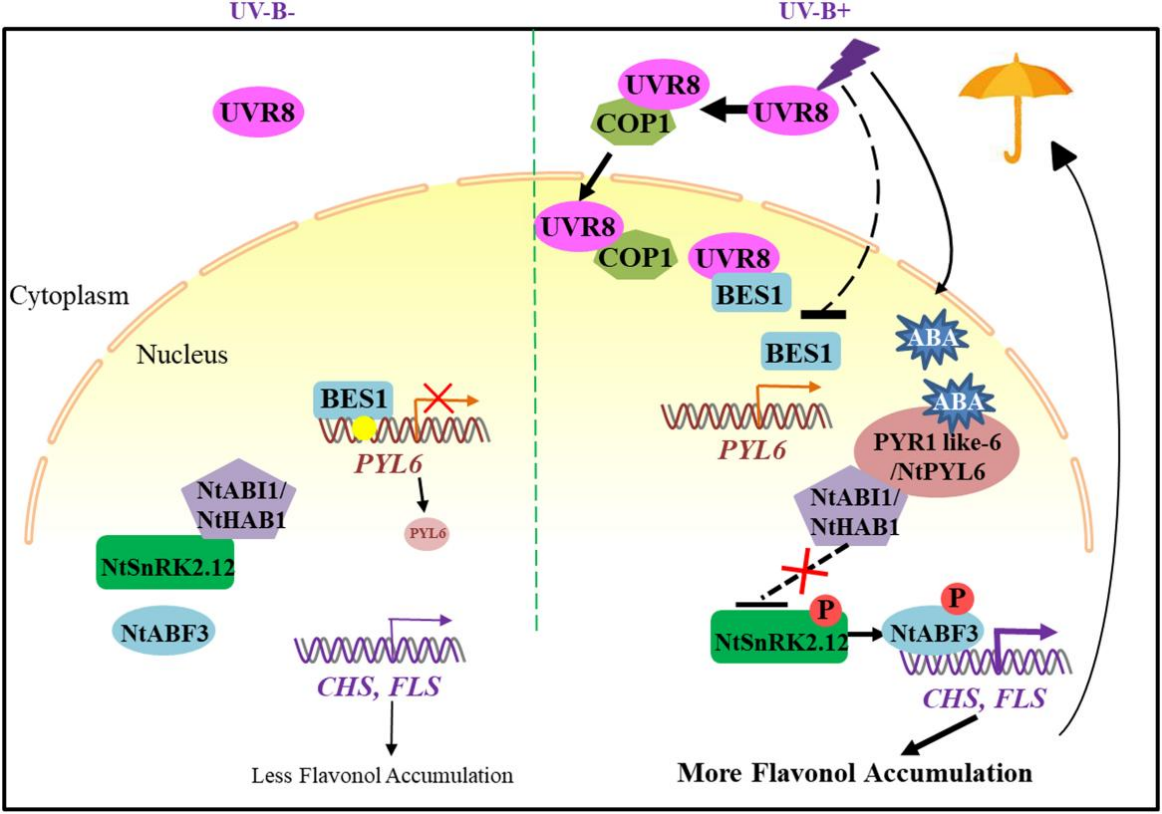
A proposed model illustrating that ABA-NtPYL6 induces flavonol accumulation to enhance plant resistance to UV-B in tobacco. When plants are grown under normal conditions, the accumulation of ABA is less, and BRI1-EMS-SUPPRESSOR1 (BES1) directly binds to the promoter of *NtPYL6* to repress its expression, leading to weak ABA signal transduction and flavonol synthesis. In response to UV-B, the biosynthesis of ABA will be enhanced and the activity of BES1 will be inhibited by UV RESISTANCE LOCUS 8 (UVR8) or other unknown factors (Liang et al. 2020), resulting in strong ABA signal transduction. In tobacco, NtPYL6-NtABI1/NtHAB1-NtSnRK2.12 constitutes the core signaling pathway, which activates the NtABF3 transcription factor. Active NtABF3 recognizes and binds to the ABREs present in the promoters of *NtCHS* and *NtFLS* genes, leading to enhanced expression of these 2 genes and increased accumulation of flavonols. Increased flavonol content helps improve plant resistance to UV-B in tobacco. COP1, CONSTITUTIVE PHOTOMORPHOGENIC 1.

Finally, we summarized our findings with a proposed regulatory network (Fig. 8). UV-B radiation induces the accumulation of ABA and the expression of *NtPYL6* leading to enhanced ABA signal. The core NtPYL6-NtABI1/NtHAB1-NtSnRK2.12 signaling pathway activates NtABF3, which recognizes and binds to the ABREs present in the promoters of *NtCHS* and *NtFLS* genes to promote their expression and the accumulation of flavonols, thereby enhancing plant resistance to UV-B. These findings confirm the roles of ABA signaling in sustaining the abilities of land plants to adapt the UV-B stress and provide strategies and targets for improving crops to resist UV-B radiation.

## Materials and methods

### Plant materials and growth conditions

Common tobacco cultivar “Honghua Dajinyuan” (HD, *Nicotiana tabacum*) seeds were germinated and grown in the greenhouse (16 h light/28 °C, 8 h darkness/23 °C) till 7 to 8 leaves. Seedlings were transplanted into the field, and leaf samples were collected from different developmental stages for gene expression analysis and metabolite detection. Rosette stage was 30 d after transplanting (DAT), flowering stage was about 60 DAT, maturation stage-1 was about 70 DAT, maturation stage-2 was about 85 DAT, and maturation stage-3 was about 100 DAT. Topping was performed at flowering stage, and leaf sample was collected 3 d after topping. The HD and *Nicotiana benthamiana* seeds were surface sterilized and germinated on 1/2 MS medium to obtain sterile seedlings (Wang et al. 2021a; He et al. 2024). These seedlings were used for ABA treatment, flavonols staining, transgenic manipulation, or transplanted into pots for other analyses.

UV-B treatment was conducted as described in our previous study (Wang et al. 2024). In brief, broadband UV-B (Philips TL 40W/12 RS SLV/25 lamps, 8 μmol/m^2^/s, measured by a UV Meter 3414F [LightScout]) was used to treat the 2-wk-old seedlings on the MS mediums for 30 min or indicated time. The possible UV-C (<280 nm) irradiation was filtered out by wrapping 0.13-mm cellulose diacetate film (Courtauld Specialty Plastics, Derby, UK) to the lamps. For different chemical treatments, approximately 2-wk-old seedlings were transferred onto new mediums containing 1 μM ABA (Zeng et al. 2022), 0.1 μM fluridone (FLD) (Tholen et al. 2007), or 100 μM rutin (Chen et al. 2019) for 3 d before UV-B treatment. After treatment, seedlings were grown under normal conditions for 1 wk and then used for photographing, calculating survival rates, and measuring different substances. After recovery, the seedlings with new normal green leaves were considered alive, while the dwarf seedlings with only yellow or dry leaves were considered dead (Wang et al. 2024). Approximately 80 seedlings were set as a replicate for survival rates calculating, and 5 independent replicates were conducted.

### Plasmid construction and plant transformation

*NtPYL6*, *NtABFs*, *NtABI1*, *NtHAB1*, and *NtSnRK2.12* all have 2 or 3 close homologous copies. For generations of different mutants with Crispr/cas9 method, the sequences of each gene including its all-homologous copies were uploaded into http://www.multicrispr.net/index.html, and common guided primers containing 20 nucleotides were designed on the website with default parameters. The primers were synthesized for the generation of sgRNAs, which were further linked to the pORE-Cas9 binary vector used for transgenic manipulation (Xie et al. 2017). For the generation of over-expression lines with fusion proteins, the *NtPYL6a* and *NtABF3a* coding sequences deleting the stop codon were amplified and fused to pCAMBIA1300-GFP and pGreen-35S-GR plasmids, respectively. Transformation of tobacco (*N. tabacum*) was performed by using the leaf disk transformation-regeneration method (Navarro-González et al. 2019). Knock-out mutants were identified using the Hi-TOM sequencing method (Liu et al. 2019; Wang et al. 2021a). Conserved primers among all the homologous copies of each gene were designed with common bridging sequences to amplify 150 to 300 bp targeted genomic DNA. The products were used to construct libraries that were sequenced by using the Illumina HiSeq platform (Illumina, USA). More than 2,000 valid reads were obtained for each gene and aligned with the reference sequences to identify whether all the homologous copies were mutated. One or 2 independent knock-out mutant lines were identified for each gene and then self-pollinated twice to obtain T_2_ homozygous mutants for subsequent analyses. At least 5 independent overexpression lines of *NtPYL6a* and *NtABF3a* were obtained, among which 3 lines with higher expression levels of target genes were selected for further analyses. The sequences of primer used for plasmid construction are shown in Table S6.

### Reverse transcription quantitative PCR (RT-qPCR) and expression analyses

A SuperPure Plantpoly RNA kit (Gene Answer, RE0220) was used to extract total RNA from different tobacco tissues. A 1-μg RNA sample was treated with DNaseI and used for the synthesis of first-strand cDNA by using Reverse Transcriptase M-MLV (Takara, 2641A). RT-qPCR was carried out on a LightCycler 96 SW 1.1 cycler (Roche). *L25* (*L25 ribosomal protein*) or *EF-1a* (*Elongation factor 1a*) was used as the reference control (Schmidt and Delaney 2010), and the gene expression levels were quantified with 2^−ΔΔCT^ or 2^−ΔCT^ method (Livak and Schmittgen 2001). The mean values of 3 independent biological replicates were considered as the gene expression levels, and the values were log2-normalized to draw the heat-maps with MEV (Multiple Experiment Viewer) software. Primers for RT-qPCR analyses are listed in Table S6.

### Broad untargeted secondary metabolome analysis

The broad untargeted secondary metabolome analysis was performed as described in our previous study (Wang et al. 2024). In brief, the metabolites of freeze-dried leaf samples were extracted with 70% methanolic buffer (L-2-chlorophenylalanine as the internal standard, 5 μmol/L). The sample extracts were then used for analysis on an UPLC-ESI-MS/MS system (https://sciex.com.cn/, UPLC, ExionLC AD) and Tandem mass spectrometry system (https://sciex.com.cn/) with previous described analytical conditions. Data acquisitions and quantifications were performed by using Analyst 1.6.3 software (Sciex) and Multiquant 3.0.3 software (Sciex). In-house R program and database (MetWare, http://www.metware.cn/) were used for peak detection and annotation. Metabolites with |Log2FC| ≥ 1.0 and *P*-value <0.05 were defined as DAMs, which were further annotated with KEGG compound database (http://www.kegg.jp/kegg/compound/). All the DAMs are listed in Table S1.

### Measurement of flavonols content

Freeze-dried tobacco tissues were ground into fine powers, and 50-mg samples were weighed. 1.5 mL 80% (v/v) ethanol extraction buffer (vitexin as internal standard, 0.012 g/L) was added to each sample, and the mixture was sonicated for 30 min. After 10 min centrifugation (14,000 rpm), the supernatant was collected through a 0.22-μm membrane and then used for the measurement of flavonols by using an HPLC-UV. The analytical conditions are as described in previous studies (Wang et al. 2019, 2020). Briefly, chromatographic column- ACQUITY UPLC BEH Phenyl (1.7 μm 2.1 mm × 150 mm, Waters); injection volume-1 μL; gas temperature-350 °C; flow-0.3 mL/min; wave length-230 nm, 260, 360, and 570 nm; mobile phase-A 100% water, B 100% acetonitrile; gradient elution-8 min 85% A + 15% B, 5 min 58% A + 42% B, 0.01 min 100% B, 3 min 95% A + 5% B, 18 min stop.

### RNA sequencing and data analysis

Qualified RNA samples were extracted and used to construct the sequencing libraries with a TruSeq RNA Sample Preparation kit (Illumina, RS-122-2001). Sequencing was carried out on the Illumina Hiseq sequencing platform. Data analysis was performed as described previously (Wang et al. 2020). Raw reads were filtered with FastQC (https://www.bioinformatics.babraham.ac.uk/projects/fastqc/) and Trimmomatic (v0.30) to obtain clean reads, which were then mapped to the reference *N. tabacum* genome (ftp://ftp.solgenomics.net/genomes/Nicotiana_tabacum/) (Edwards et al. 2017). Cufflinks (v2.2.1) were used to calculate the gene expression levels. Genes with |Log2FC| ≥ 1.0 (FDR < 0.05) were defined as DEGs. All the DEGs are shown in Tables S2 and S3.

### Induction of the GR fusion proteins

Seeds of *35S:NtPYL6a-GR* or *35S:NtABF3a-GR* lines were germinated on 1/2 MS medium containing 0.3 mg/L Basta. Approximately 2-wk-old seedlings were selected and transferred to new 1/2 MS mediums with different chemicals. The final concentration of dexamethasone (DEX) was 10 μM, and that of cycloheximide (CYC) was 5 μM. After 1-h treatment, the seedlings were collected and used for RNA extraction.

### Yeast one-hybrid (Y1H)

Putative cis-elements were searched in the 3-kb upstream sequences of *NtCHS*, *NtFLS*, *NtPAL*, *Nt4CL*, *NtLDOX*, and *NtCCoAMT1* genes. Approximately 200- to 300-bp promoter fragments containing the ABREs of these genes were amplified with specific primers and separately linked to the pLacZi2u vector. Full length CDS of *NtABF1a*, *NtABF2a*, *NtABF3a*, *NtABF4a*, *NtABF5a*, *NtABI2a*, *NtABI3a*, and *NtABI5a* were inserted into the pJG4-5 vector. The generated pJG4-5 and pLacZi2u fusion constructs were co-transformed into EGY48 yeast strain as described previously (Wang et al. 2024). The positive clones were dotted on SD/Gal/Raf/X-gal–Trp/–Ura medium for 5 to 7 d (30 °C), and photographs were then taken. The primers for YIH assay are listed in Table S6.

### Chromatin immunoprecipitation (ChIP) assay

ChIP assay was performed with the *35S:NtABF3a-GFP* seedlings, and the experimental procedure was as described previously (Wang et al. 2020). Briefly, leaves of the 2-wewkek-old seedlings grown on the 1/2 MS were collected and fixed with 1.0% formaldehyde under vacuum conditions. The samples were then ground to powders for chromatin immunoprecipitation by using an EpiQuik Chromatin Immunoprecipitation Kit (Epigentek) according to the manufacturer's introduction. DNA samples were collected and used for subsequent quantitative PCR analyses. Enrichment folds of target fragments were calculated with IP values and corresponding input values. The primers for ChIP assay are listed in Table S6.

### Electrophoretic mobility shift assay (EMSA)

The probes of *NtCHS* and *NtFLS* containing normal or mutated ABREs were synthesized by Huada Biotech (Beijing, China) (Table S5). A LightShift Chemiluminescent EMSA kit (Thermo Scientific, USA) was used to conduct the EMSA. NtABF-HIS proteins were incubated with different probes (biotin-labeled, mutated probes, and unlabeled) for 30 min and then run on a polyacrylamide gel to separate free and bound probes (Hellman and Fried 2007). Signals on Biodyne B nylon membranes (Pall Corporation, USA) were visualized with reagents in the kit and ChemiDoc XRS (Bio-Rad Laboratories, USA).

### Dual-luciferase and split-luciferase assay

For dual-luciferase assay, the reporter constructs were generated by inserting the amplified promoter fragments of target genes into pGreenII 0800-LUC to drive the expression of *LUC* gene. The effector vectors were constructed by fusing the full length CDS of different *NtABF* genes into the pGreenII 62-SK plasmids. For split-luciferase assay, CDS of target genes deleting the stop codon were amplified and inserted into the pCAMBIA1300-nLUC and pCAMBIA1300-cLUC vectors, respectively. The AtPAP1-nLUC and AtTT8-cLUC were used as positive control. All the recombined plasmids were individually transformed into the *Agrobacterium tumefaciens* strain GV3101 (pSoup-p19). Primers for vector construction are listed in Table S6.

The reporter-effector combinations of dual-luciferase assay or the nLUC-cLUC combinations of split-luciferase assay were co-injected into the leaves of *N. benthamiana*. After infection, the tobacco plants were kept in dark for 24 h and then grown under normal conditions for 2 days (Wang et al. 2023). Then 1 M luciferin (Sigma, L9504) was sprayed to the infected leaves, which were kept in dark for 5 min before photographed with a CCD (Charge Coupled Device) imaging apparatus (Bio-one, China). For dual-luciferase assay in protoplasts, the protoplasts were prepared from the leaves of different tobacco plants as described in previous study (Schweiger and Schwenkert 2014). The reporter and effector plasmids were introduced into the protoplasts via PEG-mediated method (Worley et al. 2000). For ABA treatment, protoplasts were incubated overnight at room temperature with 1 μM ABA after transformation. Relative LUC/REN activities were quantified with a Dual-Luciferase Reporter Assay System kit (Promega, E1910).

### Staining of flavonols

The flavonols in tobacco roots were stained with diphenylboric acid-2-aminoethyl ester (DPBA) as described previously (Wang et al. 2019; Nguyen 2020). Briefly, tobacco seeds were germinated on 1/2 MS which were placed vertically. One-week-old seedlings were dipped into the staining solution containing 0.25% (w/v) of DPBA and 0.2% (v/v) of Triton X-100 for 3 h. The roots were then washed with distilled water, and photographed with an epifluorescence microscope (Olympus, Tokyo, Japan). For kaempferol-DPBA, the emission spectrum was set as 475 to 500 nm, and 585 to 619 nm was set for quercetin-DPBA. The photos of kaempferol-DPBA and quercetin-DPBA were merged to indicate the content of flavonols in different tobacco roots.

### Yeast two-hybrid (Y2H)

The CDS of each couple genes needed to be detected were amplified and fused the pGADT7 and pGBKT7 vector, respectively. The recombined plasmids were co-transformed into yeast AH109 strain by using a Yeastmaker™ Yeast Transformation System 2 (Clontech, No. 630439), and the strains were incubated on SC/-Trp-Leu mediums for 3 to 4 d at 30 ℃. The positive clones were dotted on the new SC/-Trp-Leu and SC/-Leu/-Trp/-His/-Ade mediums and incubated for another 5 to 7 d before photographing.

### Measurement of chl, H_2_O_2_, O_2_^−^, and antioxidant capacities

Determination of Chl, H_2_O_2_, and O_2_^−^ contents was conducted as previously (Sun et al. 2020). Briefly, tobacco leaves were extracted with cold acetone and H_2_SO_4_ buffer, and the absorbance of the supernatant was measured 415 nm. H_2_O_2_ concentration was calculated with an established standard curve. O_2_^−^ was extracted from tobacco leaves with potassium phosphate buffer. Absorbance of the extract and NaNO_2_ solutions was measured at 530 nm and used for standard curve construction and calculation of O_2_^−^ production. For Chl measurement, leaf samples were bleached in 80% acetone, and absorbance of the extract was measured at 663 and 645 nm. The of the supernatant was recorded and 470 nm. Chlorophyll a content = 12.21 × *A_663_*− 2.81 × *A_645_*, and Chlorophyll b content = 20.13 × *A_645_*−5.03 × *A_663_*. Chlorophyll (a + b) was calculated as total chlorophyll content. Trolox equivalent antioxidant capacity (TEAC) and ferric reducing capacity (FRAP) were determined as described previously (Mátai et al. 2019).

### Statistical analysis

Statistical analysis in this study was performed as described previously (Wang et al. 2024). The values shown in the figures were means of at least 3 biological replicates with SD. All data was collected and classified with Win-Excel and analyzed with the SPSS statistical package (version 8.0). Student *t* test or Tukey test were performed as described in the legends to test the significant differences.

### Accession numbers

Sequence data and accession numbers from this article can be found in Table S5.

## Supplementary Material

kiag257_Supplementary_Data

## Data Availability

All relevant data can be found within the manuscript and its supporting materials.

## References

[kiag257-B1] Agati G, Tattini M. 2010. Multiple functional roles of flavonoids in photoprotection. New Phytol. 186:786–793. 10.1111/j.1469-8137.2010.03269.x. 20569414

[kiag257-B2] Aleman F et al 2016. An ABA-increased interaction of the PYL6 ABA receptor with MYC2 transcription factor: a putative link of ABA and JA signaling. Sci Rep. 6:28941. 10.1038/srep28941. 27357749 PMC4928087

[kiag257-B3] An JP et al 2021. ABI5 regulates ABA-induced anthocyanin biosynthesis by modulating the MYB1-bHLH3 complex in apple. J Exp Bot. 72:1460–1472. 10.1093/jxb/eraa525. 33159793

[kiag257-B4] Bai G et al 2019. Genome-wide identification and characterization of ABA receptor PYL/RCAR gene family reveals evolution and roles in drought stress in Nicotiana tabacum. BMC Genomics. 20:575. 10.1186/s12864-019-5839-2. 31296158 PMC6625023

[kiag257-B5] Berli FJ et al 2010. Abscisic acid is involved in the response of grape (Vitis vinifera L.) cv. Malbec leaf tissues to ultraviolet-B radiation by enhancing ultraviolet-absorbing compounds, antioxidant enzymes and membrane sterols. Plant Cell Environ. 33:1–10. 10.1111/j.1365-3040.2009.02044.x. 19781012

[kiag257-B6] Berli FJ, Fanzone M, Piccoli P, Bottini R. 2011. Solar UV-B and ABA are involved in phenol metabolism of Vitis vinifera L. increasing biosynthesis of berry skin polyphenols. J Agric Food Chem. 59:4874–4884. 10.1021/jf200040z. 21469737

[kiag257-B7] Bhagat PK, Verma D, Sharma D, Sinha AK. 2021. HY5 and ABI5 transcription factors physically interact to fine tune light and ABA signaling in Arabidopsis. Plant Mol Biol. 107:117–127. 10.1007/s11103-021-01187-z. 34490593

[kiag257-B8] Brunetti C, Sebastiani F, Tattini M. 2019. Review: ABA, flavonols, and the evolvability of land plants. Plant Sci. 280:448–454. 10.1016/j.plantsci.2018.12.010. 30824025

[kiag257-B9] Chen S et al 2019. NtMYB4 and NtCHS1 are critical factors in the regulation of flavonoid biosynthesis and are involved in salinity responsiveness. Front Plant Sci. 10:178. 10.3389/fpls.2019.00178. 30846995 PMC6393349

[kiag257-B10] Claeys H, Inzé D. 2013. The agony of choice: how plants balance growth and survival under water-limiting conditions. Plant Physiol. 162:1768–1779. 10.1104/pp.113.220921. 23766368 PMC3729759

[kiag257-B11] Clayton WA et al 2018. UVR8-mediated induction of flavonoid biosynthesis for UVB tolerance is conserved between the liverwort Marchantia polymorpha and flowering plants. Plant J. 96:503–517. 10.1111/tpj.14044. 30044520

[kiag257-B12] Ðinh ST, Gális I, Baldwin IT. 2013. UVB radiation and 17-hydroxygeranyllinalool diterpene glycosides provide durable resistance against mirid (Tupiocoris notatus) attack in field-grown Nicotiana attenuata plants. Plant Cell Environ. 36:590–606. 10.1111/j.1365-3040.2012.02598.x. 22897424

[kiag257-B13] Edwards KD et al 2017. A reference genome for Nicotiana tabacum enables map-based cloning of homeologous loci implicated in nitrogen utilization efficiency. BMC Genomics. 18:448. 10.1186/s12864-017-3791-6. 28625162 PMC5474855

[kiag257-B14] Fujii H et al 2009. In vitro reconstitution of an abscisic acid signalling pathway. Nature. 462:660–664. 10.1038/nature08599. 19924127 PMC2803041

[kiag257-B15] Furihata T et al 2006. Abscisic acid-dependent multisite phosphorylation regulates the activity of a transcription activator AREB1. Proc Natl Acad Sci U S A. 103:1988–1993. 10.1073/pnas.0505667103. 16446457 PMC1413621

[kiag257-B16] Gai Z et al 2020. Exogenous abscisic acid induces the lipid and flavonoid metabolism of tea plants under drought stress. Sci Rep. 10:12275. 10.1038/s41598-020-69080-1. 32704005 PMC7378251

[kiag257-B17] Gamble PE, Mullet JE. 1986. Inhibition of carotenoid accumulation and abscisic acid biosynthesis in fluridone-treated dark-grown barley. Eur J Biochem. 160:117–121. 10.1111/j.1432-1033.1986.tb09947.x. 2945718

[kiag257-B18] Han T, Wu W, Li W. 2021. Transcriptome analysis revealed the mechanism by which exogenous ABA increases anthocyanins in blueberry fruit during veraison. Front Plant Sci. 12:758215. 10.3389/fpls.2021.758215. 34858461 PMC8632357

[kiag257-B19] He S et al 2024. NtWIN1 regulates the biosynthesis of scopoletin and chlorogenic acid by targeting NtF6'H1 and NtCCoAMT genes in Nicotiana tabacum. Plant Physiol Biochem. 214:108937. 10.1016/j.plaphy.2024.108937. 39018774

[kiag257-B20] Hellman LM, Fried MG. 2007. Electrophoretic mobility shift assay (EMSA) for detecting protein-nucleic acid interactions. Nat Protoc. 2:1849–1861. 10.1038/nprot.2007.249. 17703195 PMC2757439

[kiag257-B21] Hwang K, Susila H, Nasim Z, Jung JY, Ahn JH. 2019. Arabidopsis ABF3 and ABF4 transcription factors act with the NF-YC Complex to regulate SOC1 expression and mediate drought-accelerated flowering. Mol Plant. 12:489–505. 10.1016/j.molp.2019.01.002. 30639313

[kiag257-B22] Julkowska MM, Testerink C. 2015. Tuning plant signaling and growth to survive salt. Trends Plant Sci. 20:586–594. 10.1016/j.tplants.2015.06.008. 26205171

[kiag257-B23] Kaiserli E, Jenkins GI. 2007. UV-B promotes rapid nuclear translocation of the Arabidopsis UV-B specific signaling component UVR8 and activates its function in the nucleus. Plant Cell. 19:2662–2673. 10.1105/tpc.107.053330. 17720867 PMC2002606

[kiag257-B24] Kobayashi Y et al 2005. Abscisic acid-activated SNRK2 protein kinases function in the gene-regulation pathway of ABA signal transduction by phosphorylating ABA response element-binding factors. Plant J. 44:939–949. 10.1111/j.1365-313X.2005.02583.x. 16359387

[kiag257-B25] Lai B et al 2014. LcMYB1 is a key determinant of differential anthocyanin accumulation among genotypes, tissues, developmental phases and ABA and light stimuli in Litchi chinensis. PLoS One. 9:e86293. 10.1371/journal.pone.0086293. 24466010 PMC3897698

[kiag257-B26] Li G et al 2019. ABA mediates development-dependent anthocyanin biosynthesis and fruit coloration in Lycium plants. BMC Plant Biol. 19:317. 10.1186/s12870-019-1931-7. 31307384 PMC6631627

[kiag257-B27] Li J, Ou-Lee TM, Raba R, Amundson RG, Last RL. 1993. Arabidopsis flavonoid mutants are hypersensitive to UV-B irradiation. Plant Cell. 5:171–179. 10.2307/3869583. 12271060 PMC160260

[kiag257-B28] Li L et al 2009. Arabidopsis MYB30 is a direct target of BES1 and cooperates with BES1 to regulate brassinosteroid-induced gene expression. Plant J. 58:275–286. 10.1111/j.1365-313X.2008.03778.x. 19170933 PMC2814797

[kiag257-B29] Liang T et al 2018. UVR8 interacts with BES1 and BIM1 to regulate transcription and photomorphogenesis in Arabidopsis. Dev Cell. 44:512–523.e5. 10.1016/j.devcel.2017.12.028. 29398622

[kiag257-B30] Liang T et al 2020. Brassinosteroid-activated BRI1-EMS-SUPPRESSOR 1 inhibits flavonoid biosynthesis and coordinates growth and UV-B stress responses in plants. Plant Cell. 32:3224–3239. 10.1105/tpc.20.00048. 32796123 PMC7534464

[kiag257-B31] Liang T, Yang Y, Liu H. 2019. Signal transduction mediated by the plant UV-B photoreceptor UVR8. New Phytol. 221:1247–1252. 10.1111/nph.15469. 30315741

[kiag257-B32] Liu Q et al 2019. Hi-TOM: a platform for high-throughput tracking of mutations induced by CRISPR/Cas systems. Sci China Life Sci. 62:1–7. 10.1007/s11427-018-9402-9. 30446870

[kiag257-B33] Liu WC et al 2022. Coordination of plant growth and abiotic stress responses by tryptophan synthase β subunit 1 through modulation of tryptophan and ABA homeostasis in Arabidopsis. Mol Plant. 15:973–990. 10.1016/j.molp.2022.04.009. 35488429

[kiag257-B34] Livak KJ, Schmittgen TD. 2001. Analysis of relative gene expression data using real-time quantitative PCR and the 2(-Delta Delta C(T)). Method. Methods. 25:402–408. 10.1006/meth.2001.1262. 11846609

[kiag257-B35] Loreti E et al 2008. Gibberellins, jasmonate and abscisic acid modulate the sucrose-induced expression of anthocyanin biosynthetic genes in Arabidopsis. New Phytol. 179:1004–1016. 10.1111/j.1469-8137.2008.02511.x. 18537890

[kiag257-B36] Mátai A, Nagy D, Hideg É. 2019. UV-B strengthens antioxidant responses to drought in Nicotiana benthamiana leaves not only as supplementary irradiation but also as pre-treatment. Plant Physiol Biochem. 134:9–19. 10.1016/j.plaphy.2018.09.014. 30224262

[kiag257-B37] Navarro-González SS et al 2019. Enhanced tolerance against a fungal pathogen and insect resistance in transgenic tobacco plants overexpressing an endochitinase gene from Serratia marcescens. Int J Mol Sci. 20:3482. 10.3390/ijms20143482. 31315176 PMC6679225

[kiag257-B38] Nguyen NH . 2020. A protocol for flavonols, kaempferol and quercetin, staining in plant root tips. Bio Protoc. 10:e3781. 10.21769/BioProtoc.3781. PMC7842797 33659437

[kiag257-B39] Nolan TM, Vukašinović N, Liu D, Russinova E, Yin Y. 2020. Brassinosteroids: multidimensional regulators of plant growth, development, and stress responses. Plant Cell. 32:295–318. 10.1105/tpc.19.00335. 31776234 PMC7008487

[kiag257-B40] Pan WS, Zheng LP, Tian H, Li WY, Wang JW. 2014. Transcriptome responses involved in artemisinin production in Artemisia annua L. under UV-B radiation. J Photochem Photobiol B. 140:292–300. 10.1016/j.jphotobiol.2014.08.013. 25194528

[kiag257-B41] Park SY et al 2009. Abscisic acid inhibits type 2C protein phosphatases via the PYR/PYL family of START proteins. Science. 324:1068–1071. 10.1126/science.1173041. 19407142 PMC2827199

[kiag257-B42] Peng Q, Zhou Q. 2009. The endogenous hormones in soybean seedlings under the joint actions of rare earth element La(III) and ultraviolet-B stress. Biol Trace Elem Res. 132:270–277. 10.1007/s12011-009-8404-z. 19462161

[kiag257-B43] Qi T et al 2011. The Jasmonate-ZIM-domain proteins interact with the WD-Repeat/bHLH/MYB complexes to regulate Jasmonate-mediated anthocyanin accumulation and trichome initiation in Arabidopsis thaliana. Plant Cell. 23:1795–1814. 10.1105/tpc.111.083261. 21551388 PMC3123955

[kiag257-B44] Rizzini L et al 2011. Perception of UV-B by the Arabidopsis UVR8 protein. Science. 332:103–106. 10.1126/science.1200660. 21454788

[kiag257-B45] Rozema J, van de Staaij J, Björn LO, Caldwell M. 1997. UV-B as an environmental factor in plant life: stress and regulation. Trends Ecol Evol. 12:22–28. 10.1016/S0169-5347(96)10062-8. 21237957

[kiag257-B46] Schmidt GW, Delaney SK. 2010. Stable internal reference genes for normalization of real-time RT-PCR in tobacco (Nicotiana tabacum) during development and abiotic stress. Mol Genet Genomics. 283:233–241. 10.1007/s00438-010-0511-1. 20098998

[kiag257-B47] Schweiger R, Schwenkert S. 2014. Protein-protein interactions visualized by bimolecular fluorescence complementation in tobacco protoplasts and leaves. J Vis Exp. 85:51327. 10.3791/51327. PMC4144666 24637460

[kiag257-B48] Shi L, Lin K, Su T, Shi F. 2023. Abscisic acid inhibits cortical microtubules reorganization and enhances ultraviolet-B tolerance in Arabidopsis thaliana. Genes (Basel). 14:892. 10.3390/genes14040892. 37107650 PMC10137628

[kiag257-B49] Song Z et al 2022. F-box protein EBF1 and transcription factor ABI5-like regulate banana fruit chilling-induced ripening disorder. Plant Physiol. 188:1312–1334. 10.1093/plphys/kiab532. 34791491 PMC8825429

[kiag257-B50] Stracke R et al 2007. Differential regulation of closely related R2R3-MYB transcription factors controls flavonol accumulation in different parts of the Arabidopsis thaliana seedling. Plant J. 50:660–677. 10.1111/j.1365-313X.2007.03078.x. 17419845 PMC1976380

[kiag257-B51] Stracke R et al 2010. The Arabidopsis bZIP transcription factor HY5 regulates expression of the PFG1/MYB12 gene in response to light and ultraviolet-B radiation. Plant Cell Environ. 33:88–103. 10.1111/j.1365-3040.2009.02061.x. 19895401

[kiag257-B52] Sun H et al 2020. MaCDSP32 from mulberry enhances resilience post-drought by regulating antioxidant activity and the osmotic content in transgenic tobacco. Front Plant Sci. 11:419. 10.3389/fpls.2020.00419. 32373141 PMC7177052

[kiag257-B53] Tholen D, Pons TL, Voesenek LA, Poorter H. 2007. Ethylene insensitivity results in down-regulation of rubisco expression and photosynthetic capacity in tobacco. Plant Physiol. 144:1305–1315. 10.1104/pp.107.099762. 17535822 PMC1914117

[kiag257-B54] Tossi V, Lamattina L, Cassia R. 2009. An increase in the concentration of abscisic acid is critical for nitric oxide-mediated plant adaptive responses to UV-B irradiation. New Phytol. 181:871–879. 10.1111/j.1469-8137.2008.02722.x. 19140950

[kiag257-B55] Vanhaelewyn L, Prinsen E, Van Der Straeten D, Vandenbussche F. 2016. Hormone-controlled UV-B responses in plants. J Exp Bot. 67:4469–4482. 10.1093/jxb/erw261. 27401912

[kiag257-B56] Wang M, Wang Y, Xie C, Wang P, Yang R. 2025. The regulation of UV-B—triggered ABA signal on isoflavones synthesis in soybean suspension cells. Plant Physiol Biochem. 222:109728. 10.1016/j.plaphy.2025.109728. 40048945

[kiag257-B57] Wang Z et al 2019. Evolutionary and functional analyses of the 2-oxoglutarate-dependent dioxygenase genes involved in the flavonoid biosynthesis pathway in tobacco. Planta. 249:543–561. 10.1007/s00425-018-3019-2. 30293202

[kiag257-B58] Wang Z et al 2020. Functional characterization of a HD-ZIP IV transcription factor NtHDG2 in regulating flavonols biosynthesis in Nicotiana tabacum. Plant Physiol Biochem. 146:259–268. 10.1016/j.plaphy.2019.11.033. 31778931

[kiag257-B59] Wang Z et al 2021a. NtMYB12a acts downstream of sucrose to inhibit fatty acid accumulation by targeting lipoxygenase and SFAR genes in tobacco. Plant Cell Environ. 44:775–791. 10.1111/pce.13957. 33225450

[kiag257-B60] Wang Z et al 2021b. Molecular cloning and functional characterization of NtWRKY11b in promoting the biosynthesis of flavonols in Nicotiana tabacum. Plant Sci. 304:110799. 10.1016/j.plantsci.2020.110799. 33568298

[kiag257-B61] Wang Z et al 2023. The transcription factor NtERF13a enhances abiotic stress tolerance and phenylpropanoid compounds biosynthesis in tobacco. Plant Sci. 334:111772. 10.1016/j.plantsci.2023.111772. 37331634

[kiag257-B62] Wang Z et al 2024. NtMYB27 acts downstream of NtBES1 to modulate flavonoids accumulation in response to UV-B radiation in tobacco. Plant J. 119:2867–2884. 10.1111/tpj.16958. 39133822

[kiag257-B63] Watkins JM, Chapman JM, Muday GK. 2017. Abscisic acid-induced reactive oxygen Species are modulated by flavonols to control stomata aperture. Plant Physiol. 175:1807–1825. 10.1104/pp.17.01010. 29051198 PMC5717730

[kiag257-B64] Worley CK et al 2000. Degradation of Aux/IAA proteins is essential for normal auxin signalling. Plant J. 21:553–562. 10.1046/j.1365-313x.2000.00703.x. 10758506

[kiag257-B65] Xian B et al 2024. The ABI4-RGL2 module serves as a double agent to mediate the antagonistic crosstalk between ABA and GA signals. New Phytol. 241:2464–2479. 10.1111/nph.19533. 38287207

[kiag257-B66] Xie X et al 2017. Analysis of Nicotiana tabacum PIN genes identifies NtPIN4 as a key regulator of axillary bud growth. Physiol Plant. 160:222–239. 10.1111/ppl.12547. 28128458

[kiag257-B67] Yao X et al 2022. Brassinosteroids enhance BES1-required thermomemory in Arabidopsis thaliana. Plant Cell Environ. 45:3492–3504. 10.1111/pce.14444. 36130868

[kiag257-B68] Yoshida T et al 2015. Four Arabidopsis AREB/ABF transcription factors function predominantly in gene expression downstream of SnRK2 kinases in abscisic acid signalling in response to osmotic stress. Plant Cell Environ. 38:35–49. 10.1111/pce.12351. 24738645 PMC4302978

[kiag257-B69] Yu W, Gong F, Xu H, Zhou X. 2024. Molecular mechanism of exogenous ABA to enhance UV-B resistance in Rhododendron chrysanthum pall. By modulating flavonoid accumulation. Int J Mol Sci. 25:5248. 10.3390/ijms25105248. 38791294 PMC11121613

[kiag257-B70] Yu W, Zhou X, Meng J, Zhou X, Xu H. 2025. Multi-omics research reveals the effects of the ABA-regulated phenylpropanoid biosynthesis pathway on the UV-B response in Rhododendron chrysanthum pall. Plants (Basel). 14:101. 10.3390/plants14010101. 39795361 PMC11723134

[kiag257-B71] Yu X et al 2011. A brassinosteroid transcriptional network revealed by genome-wide identification of BESI target genes in Arabidopsis thaliana. Plant J. 65:634–646. 10.1111/j.1365-313X.2010.04449.x. 21214652

[kiag257-B72] Zeng Z, Lyu T, Lyu Y. 2022. LoSWEET14, a sugar transporter in lily, is regulated by transcription factor LoABF2 to participate in the ABA signaling pathway and enhance tolerance to multiple abiotic stresses in tobacco. Int J Mol Sci. 23:15093. 10.3390/ijms232315093. 36499419 PMC9739489

[kiag257-B73] Zhao Y et al 2014. The ABA receptor PYL8 promotes lateral root growth by enhancing MYB77-dependent transcription of auxin-responsive genes. Sci Signal. 7:ra53. 10.1126/scisignal.2005051. 24894996 PMC4298826

[kiag257-B74] Zhao Y et al 2016. ABA receptor PYL9 promotes drought resistance and leaf senescence. Proc Natl Acad Sci U S A. 113:1949–1954. 10.1073/pnas.1522840113. 26831097 PMC4763734

[kiag257-B75] Zhu JK . 2016. Abiotic stress signaling and responses in plants. Cell. 167:313–324. 10.1016/j.cell.2016.08.029. 27716505 PMC5104190

