# Supplementary material for: The ABA receptor NtPYL6 promotes flavonol biosynthesis to enhance tobacco resistance to UV-B
Source: Plant Physiol. 2026 Apr 29;201(2):kiag257. doi: 10.1093/plphys/kiag257 (PMC13281938; doi:10.1093/plphys/kiag257)
Supplement: kiag257_Supplementary_Data [file kiag257_supplementary_data.zip › Supplementary Data.pdf]

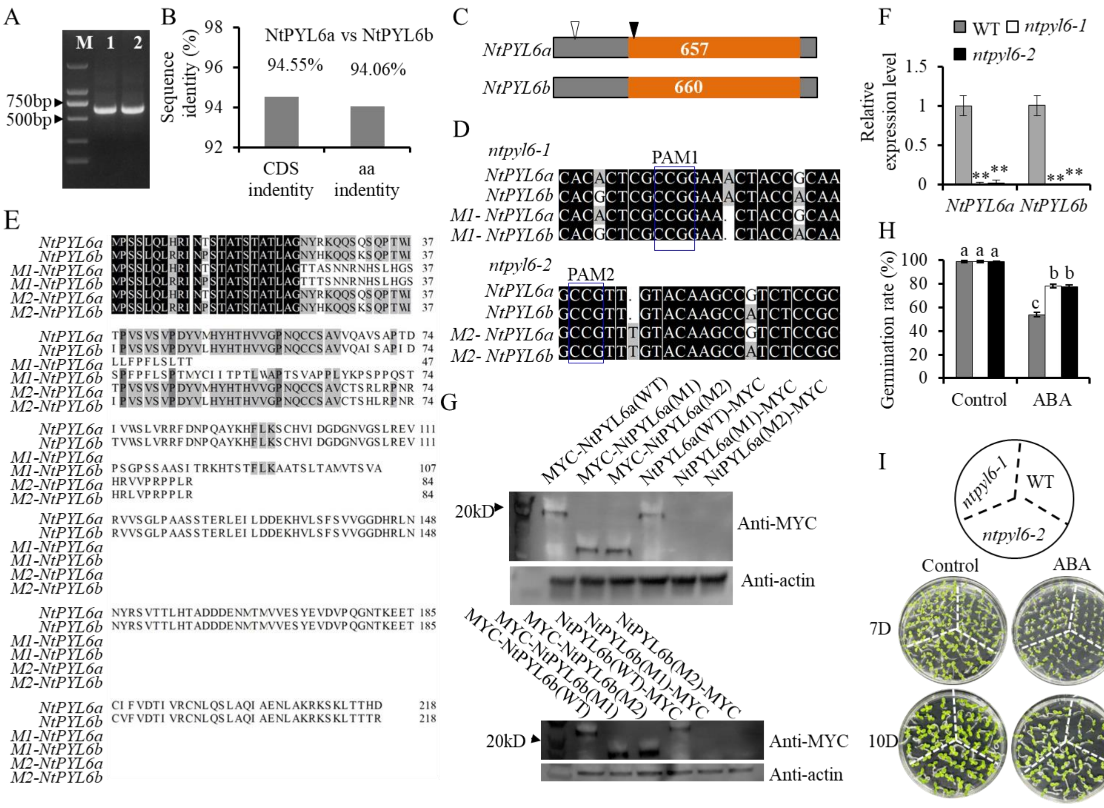

Supplementary Figure S1. Cloning of *NtPYL6* genes and generation of *ntpyl6* mutants. A, Cloning of *NtPYL6* genes from HD cultivar (*N. tabacum*) . M, DNA marker. Line 1 and 2 showed the PCR products amplified with DNA and cDNA as the template, respectively. B, Sequence identities between *NtPYL6a* and *NtPYL6b*. The CDS and amino acid sequences of *NtPYL6a* and *NtPYL6b* were separately aligned to calculate the sequence identities. C, Gene structures of *NtPYL6a* and *NtPYL6b*. Gene exon/intron structures were determined by aligning the sequenced genomic DNA and the CDS of *NtPYL6a* and *NtPYL6b*. The numbers represent the full length of *NtPYL6a* and *NtPYL6b*, respectively. The orange boxes indicate the putative Pfam domains. The triangles show the location of sgRNAs for Crispr/cas9. D, Different knock-out lines of *NtPYL6* identified by Hi-TOM sequencing. Blue boxes indicate the PAMs. Both *NtPYL6a* and *NtPYL6b* were sequenced in each line to identify the *ntpyl6* mutants. E, The putative amino acid sequences of *NtPYL6a* and *NtPYL6b* in WT and two *ntpyl6* mutant lines. F, Relative expression levels of *NtPYL6a* and *NtPYL6b* in *ntpyl6* mutants. Values are means of three independent replicates ±SD. Asterisks indicate the significant differences, detected via two-tailed paired Student's *t*-test (**,

$p < 0.01$). G, Abundance of different NtPYL6 proteins. Wild-type and mutated CDS of *NtPYL6a* and *NtPYL6b* were synthesized and fused into different vectors with MYC tag. The MYC tags were labeled either at the *N*-terminal or *C*-terminal of the target proteins. All the fused plasmids were transiently expressed in *N. benthamiana* leaves, and total protein samples were extracted from the leaves and used for western blotting analysis with anti-MYC antibody. WT indicates the CDS of wild-type plants; M1 indicates the CDS of *NtPYL6a/b* in *ntpyl6-1*; M2 indicates the CDS of *NtPYL6a/b* in *ntpyl6-2*. H, Germination rates of WT and *ntpyl6* seeds under control and ABA treatment. Values are means of five independent replicates ±SD. The significant differences are detected by Tukey's test ($p < 0.05$) and shown with letters. I, The *ntpyl6* seedlings showed reduced sensitivity to ABA treatment compared to WT seedlings. Seeds were germinated on control or ABA (0.1 μM) mediums, and photographs were taken 7 days or 10 days after germination.

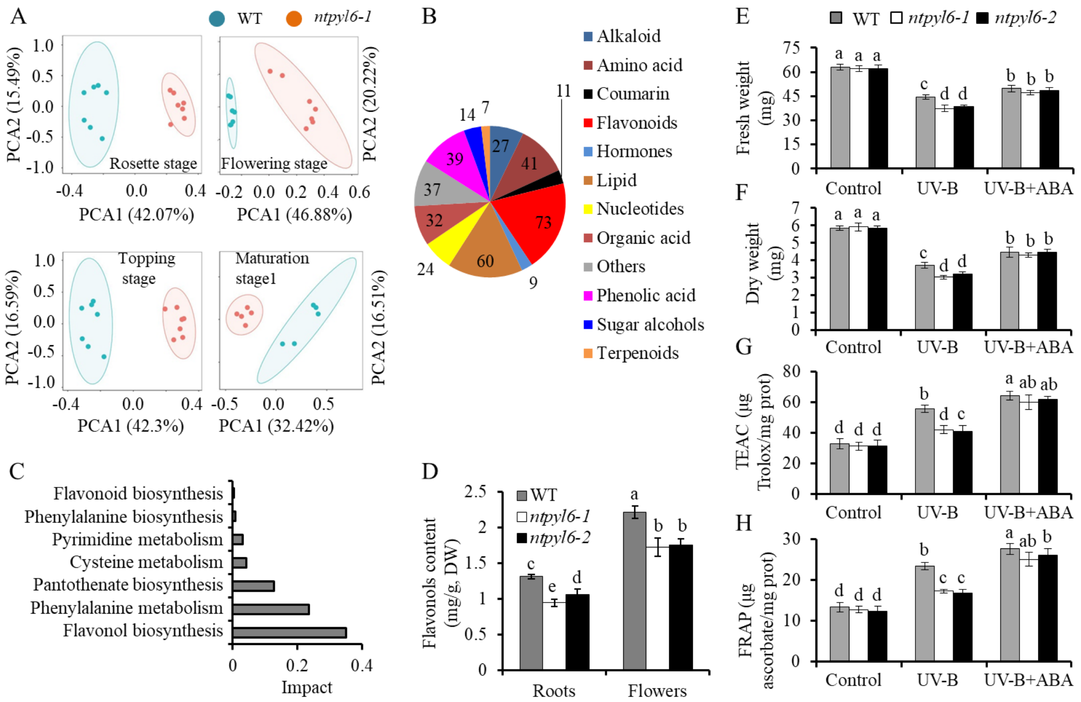

Supplementary Figure S2. Secondary metabolome analysis of WT and *ntpyl6-1* leaves. A, PCA analyses revealed different secondary metabolomes between WT and *ntpyl6-1* mutant. B, Categorization of the differentially accumulated metabolites (DAM) between WT and *ntpyl6-1* mutant. C, Analysis of the differential metabolic

pathways between WT and *ntpyl6-1* mutant. Differential metabolic pathway analysis was performed and presented by using the representative maturation-stage samples. D, The flavonol contents in the roots and flowers of WT, *ntpyl6-1*, and *ntpyl6-2* plants. Tobacco tissues were collected at the flowering stage. Values are means of three independent replicates ±SD. E and F, Fresh and dry weight of the above ground parts of WT, *ntpyl6-1*, and *ntpyl6-2* seedlings under different treatments. Values are means ±SD of ten independent replicates. G and H, Total antioxidant capacities (TEAC and FRAP) of the WT, *ntpyl6-1*, and *ntpyl6-2* seedlings under different treatments. Values are means ±SD of five independent replicates. The significant differences are detected by Tukey's test ($p < 0.05$) and shown with letters.

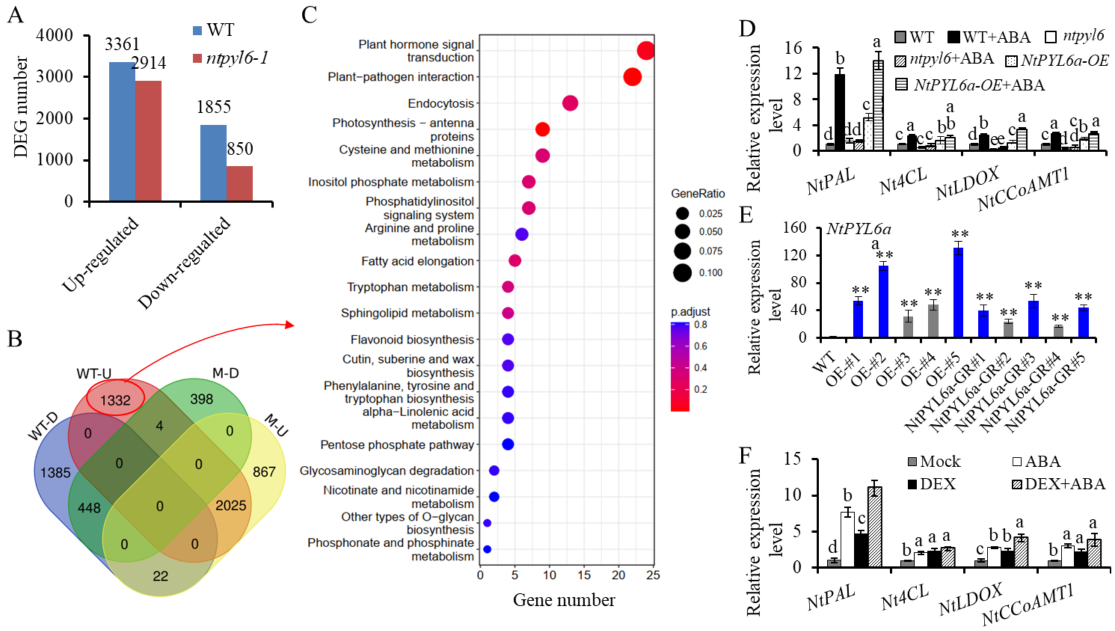

Supplementary Figure S3. RNA-seq analysis of WT and *ntpyl6-1* plants between control and ABA treatment. A, Numbers of differentially expressed genes (DEGs) in WT and *ntpyl6-1* leaves treated with or without ABA. Tobacco plants about 50 DAT were sprayed with 0.1 µM ABA every 4 hours for three days, and the 15th leaves were collected for RNA-seq analysis. Three independent biological replicates were set for each sample. B, Venn analysis between the DEGs of WT and *ntpyl6-1* plants. WT-D, the down-regulated genes by ABA treatment in WT plants; WT-U, the up-regulated genes by ABA treatment in WT plants; M-D, the down-regulated genes by ABA

treatment in the mutant (*ntpyl6-1*) plants; M-U, the up-regulated genes by ABA treatment in the mutant (*ntpyl6-1*) plants. C, KEGG annotation of the 1,322 DEGs that were significantly up-regulated in WT by ABA, but showed no significant changes in *ntpyl6-1* plants after ABA treatment. D, Relative expression levels of *NtPAL*, *Nt4CL*, *NtLDOX*, and *NtCCoAMT1* in WT, *ntpyl6*, and *NtPYL6a-OE* plants treated with or without ABA. Values are means of three independent replicates ±SD. The significant differences are detected by Tukey's test ($p < 0.05$) and shown with letters. E, Identification of *35S:NtPYL6a-GFP* and *35S:NtPYL6a-GR* transgenic lines. The blue columns represent the lines used for subsequent analysie. Values are means of three independent replicates ±SD. Asterisks indicate the significant differences, which are detected by two-tailed paired Student's t-test (**, $p < 0.01$). F, Relative expression levels of *NtPAL*, *Nt4CL*, *NtLDOX*, and *NtCCoAMT1* in *35S:NtPYL6a-GR* plants. Values are means of three independent replicates ±SD. The significant differences are detected by Tukey's test ($p < 0.05$) and shown with letters.

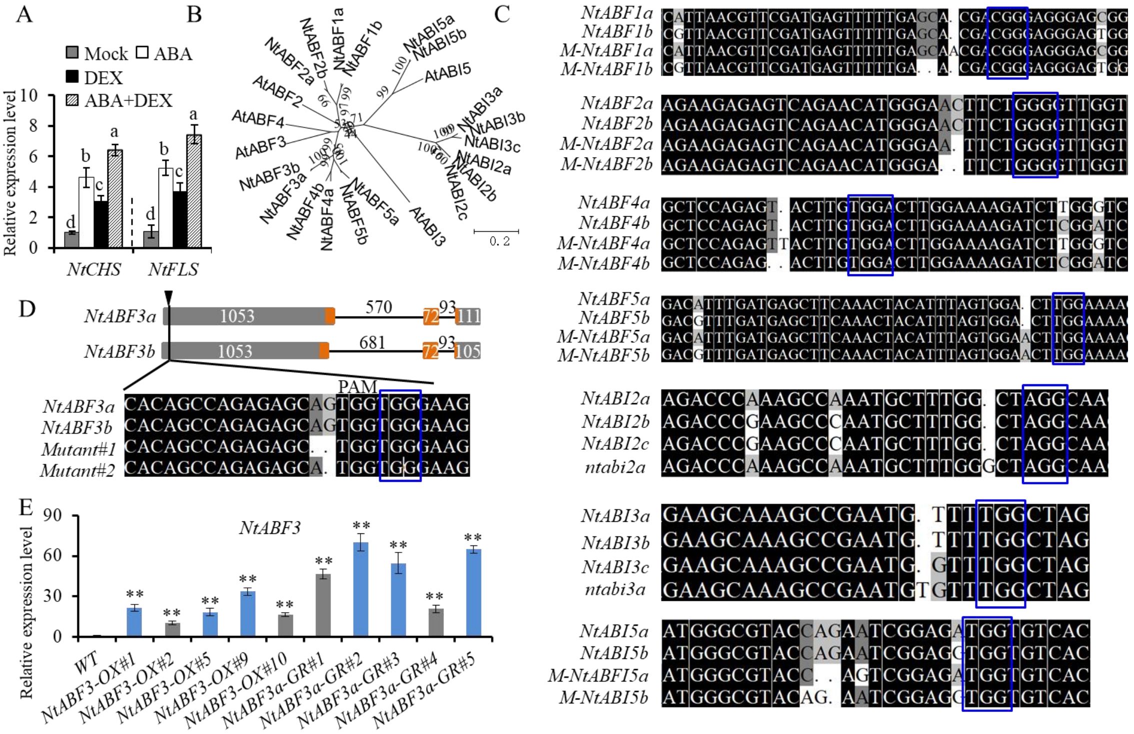

Supplementary Figure S4. Identification of different *ntabf* mutants and the *NtABF3a* overexpression plants. A, Relative expression levels of *NtCHS* and *NtFLS*

in *35S:NtPYL6a-GR* plants. Values are means of three independent replicates ±SD. The significant differences are detected by Tukey's test (p < 0.05) and shown with letters. B, Phylogenetic analysis of NtABF proteins. MEGA 5.0 software and neighbor-joining algorithm were adopted to construct the phylogenetic tree. The bar in the bottom right corner indicates the relative divergence of the sequences examined. C, Knock-out lines of different *NtABF* genes identified by Hi-TOM sequencing. Blue boxes indicate the PAMs. All the close homologous copies of each gene were sequenced in each line to identify the *ntabf* mutants. D, Gene structures of *NtABF3a/3b*, and identification of *NtABF3* knock-out lines. The numbers indicate the length (bp) of exons or introns. The orange boxes indicate the putative Pfam domains. The triangles show the location of sgRNA for Crispr/ cas9. E, Identification of *35S:NtABF3a-GFP* and *35S:NtABF36a-GR* transgenic lines. Values are means of three independent replicates ±SD. Asterisks indicate the significant differences, which are detected by two-tailed paired Student's t-test (**, p < 0.01). Three independent lines with blue color were selected for subsequent analyses.

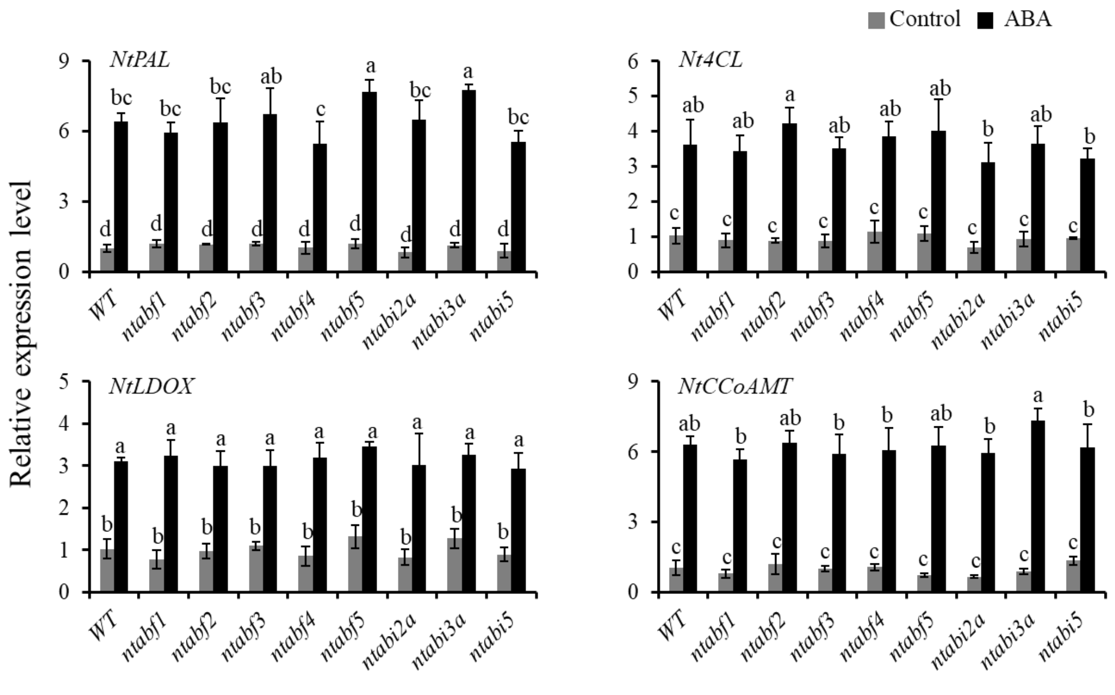

Supplementary Figure S5. Relative expression levels of four flavonol related DEGs in WT and different *ntabf* mutants. Four-week-old tobacco seedlings grown

on the 1/2 MS medium were transferred to new mediums without or with 0.1 μM ABA for 1h, and then collected for gene expression analysis. Values are means of three independent replicates ±SD. The significant differences are detected by Tukey's test ($p < 0.05$) and shown with letters.

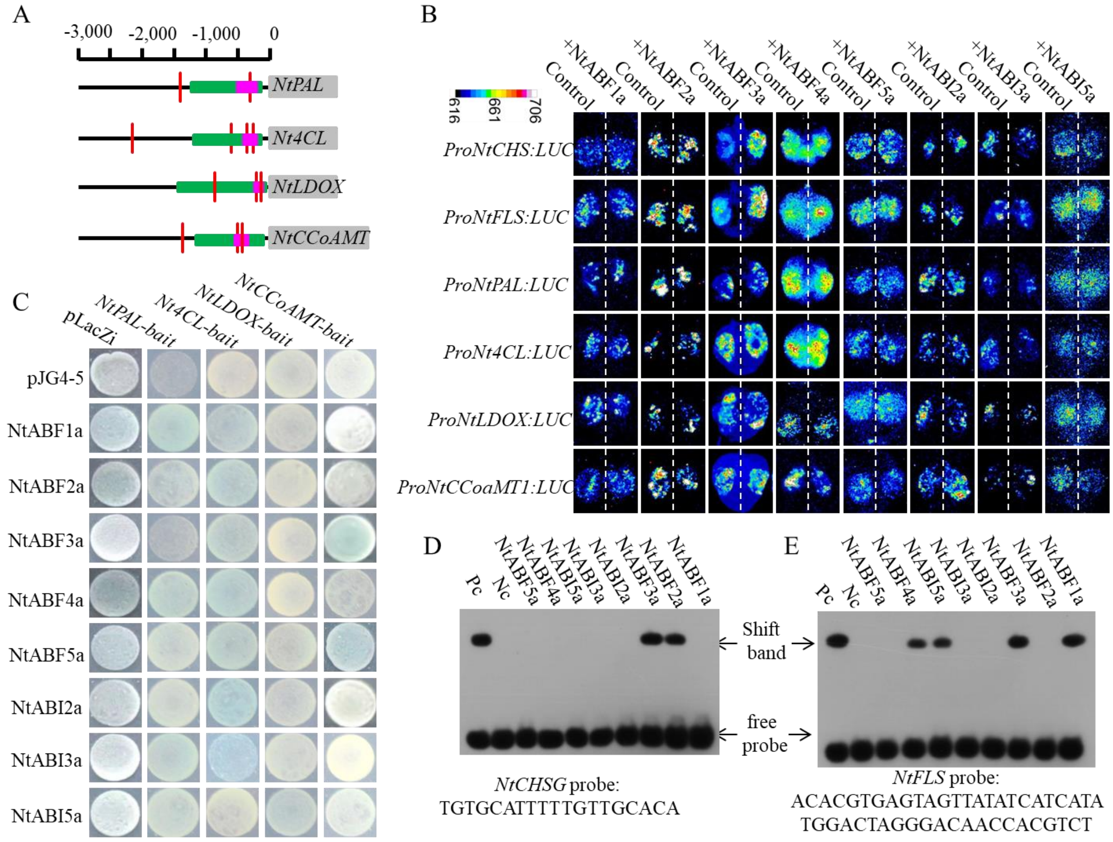

Supplementary Figure S6. The binding test of NtABFs to the flavonol related DEGs. A, Distribution of putative ABREs present in the upstream 3kb sequences of the flavonol related DEGs. Red lines represent the putative ABREs. Purple boxes indicate the fragments used for Y1H assay. Green boxes indicate the fragments used for dual-LUC assay. B, Dual-LUC assay between NtABFs and flavonol related DEGs. The images shown in Figure 5C are also shown here. The color scale bar indicates the gray value. C, Y1H assay showed no binding of NtABFs to the fragment of *NtPAL*, *Nt4CL*, *NtLDOX*, and *NtCCoAMT* genes. The pLacZi images shown in Figure 5B are the same as those shown here. D and E, EMSA revealed the in vitro binding of several NtABFs to the *NtCHS* and *NtFLS* promoters.

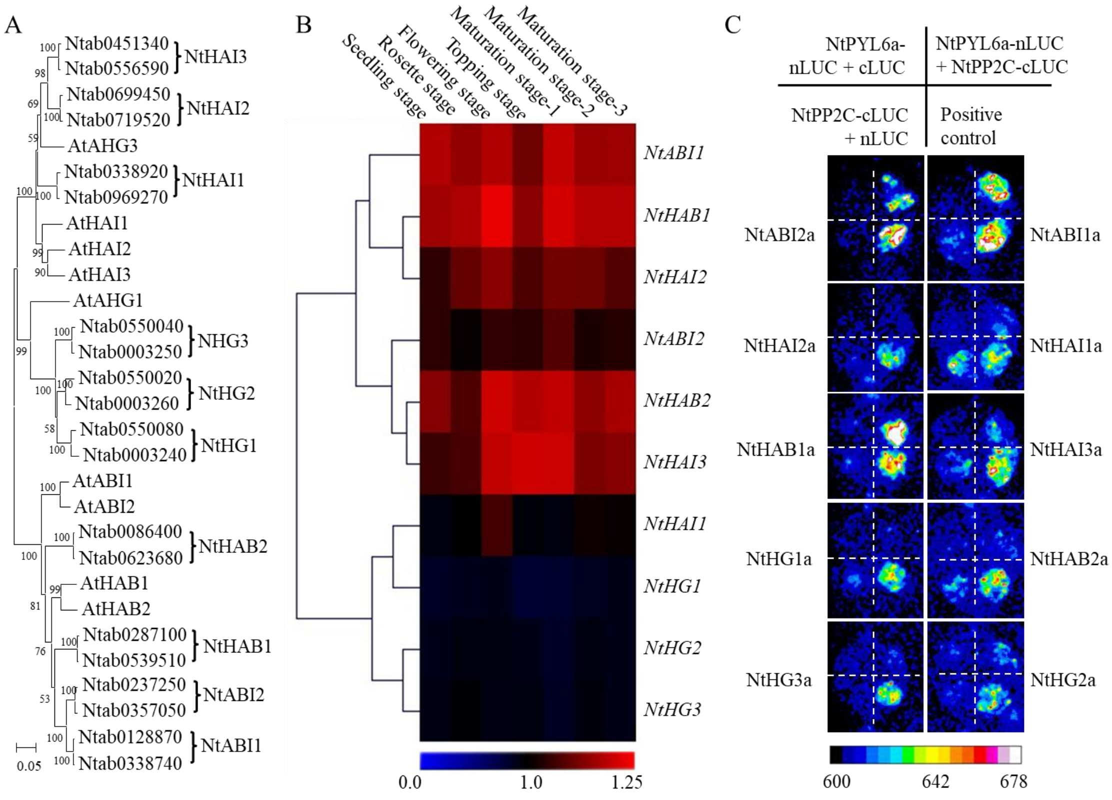

Supplementary Figure S7. Identification of the key PP2C members that interact with NtPYL6a in tobacco. A, Phylogenetic analysis of tobacco NtPP2Cs with the AtPP2Cs in Arabidopsis. The bar at the bottom indicates the relative divergence of the sequences examined. The accession number and sequence information of all the PP2Cs are shown in Supplementary Table S5. B, Expression pattern of *NtPP2C* genes in tobacco leaves from different developmental stages. The color scale bar indicates the values of log2 (gene expression level). The target gene expression levels were calculated by using $2^{-\Delta Ct}$ method with *NtL25* as reference gene. C, Split-LUC indicated that NtPYL6a interacted with NtABI1a and NtHAB1a, respectively. The color scale bar indicates the gray value.

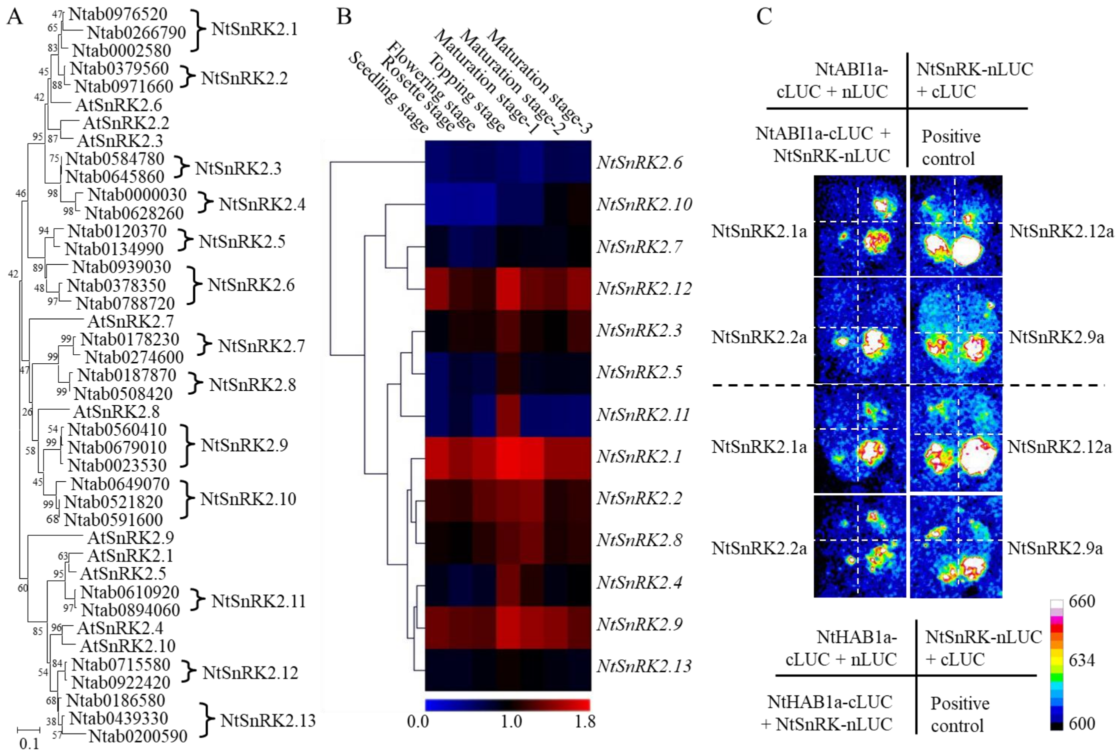

Supplementary Figure S8. Identification of the key SnRK2 members that interact with NtABI1a and NtHAB1a in tobacco. A, Phylogenetic analysis of tobacco NtSnRK2 with the AtSnRK2 in Arabidopsis. The bar at the bottom indicates the relative divergence of the sequences examined. The accession number and sequence information of all the SnRK2 are shown in Supplementary Table S5. B, Expression pattern of *NtSnRK2* genes in tobacco leaves from different developmental stages. The color scale bar indicates the values of log2 (gene expression level). The target gene expression levels were calculated by using $2^{-\Delta Ct}$ method with *NtL25* as reference gene. C, Split-LUC indicated that NtSnRK2.12 interacted with NtABI1a and NtHAB1a, respectively. The color scale bar indicates the gray value.

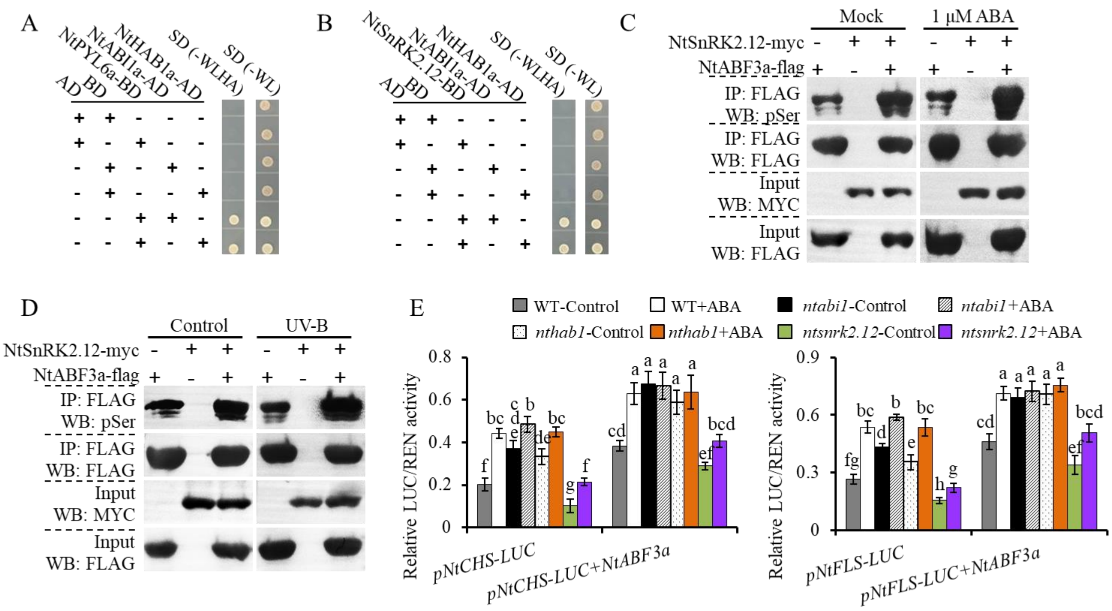

Supplementary Figure S9. The key PP2C and SnRK2 members affect the activities of NtABF3. A, Y2H assay showed that NtPYL6a interacted with NtABI1a and NtHAB1a. B, Y2H assay showed that NtSnRK2.12 interacted with NtABI1a and NtHAB1a. C and D, ABA and UV-B enhanced the phosphorylation of NtABF3a caused by NtSnRK2.12 in vivo. The NtABF3a-flag proteins were expressed alone or with NtSnRK2.12-myc in the leaves of *Nicotiana benthamiana*. Anti-FLAG and anti-MYC were used to perform western blot (WB) to confirm the existence of fusion proteins in the leaves (Input samples). Immunoprecipitation (IP) of the NtABF3a-flag proteins was conducted by using anti-FLAG antibody, and subsequent immunoblotting was performed with anti-FLAG and anti-pSer/pThr antibodies. E, LUC intensities of *pNtCHS-LUC* and *pNtFLS-LUC* co-injected with *NtABF3a* or not into the protoplasts of WT, *ntabi1*, *nthab1*, and *ntsnrk2.12* treated with or without ABA. Values are means of three independent replicates ±SD. The significant differences are detected by Tukey's test ($p < 0.05$) and shown with letters.

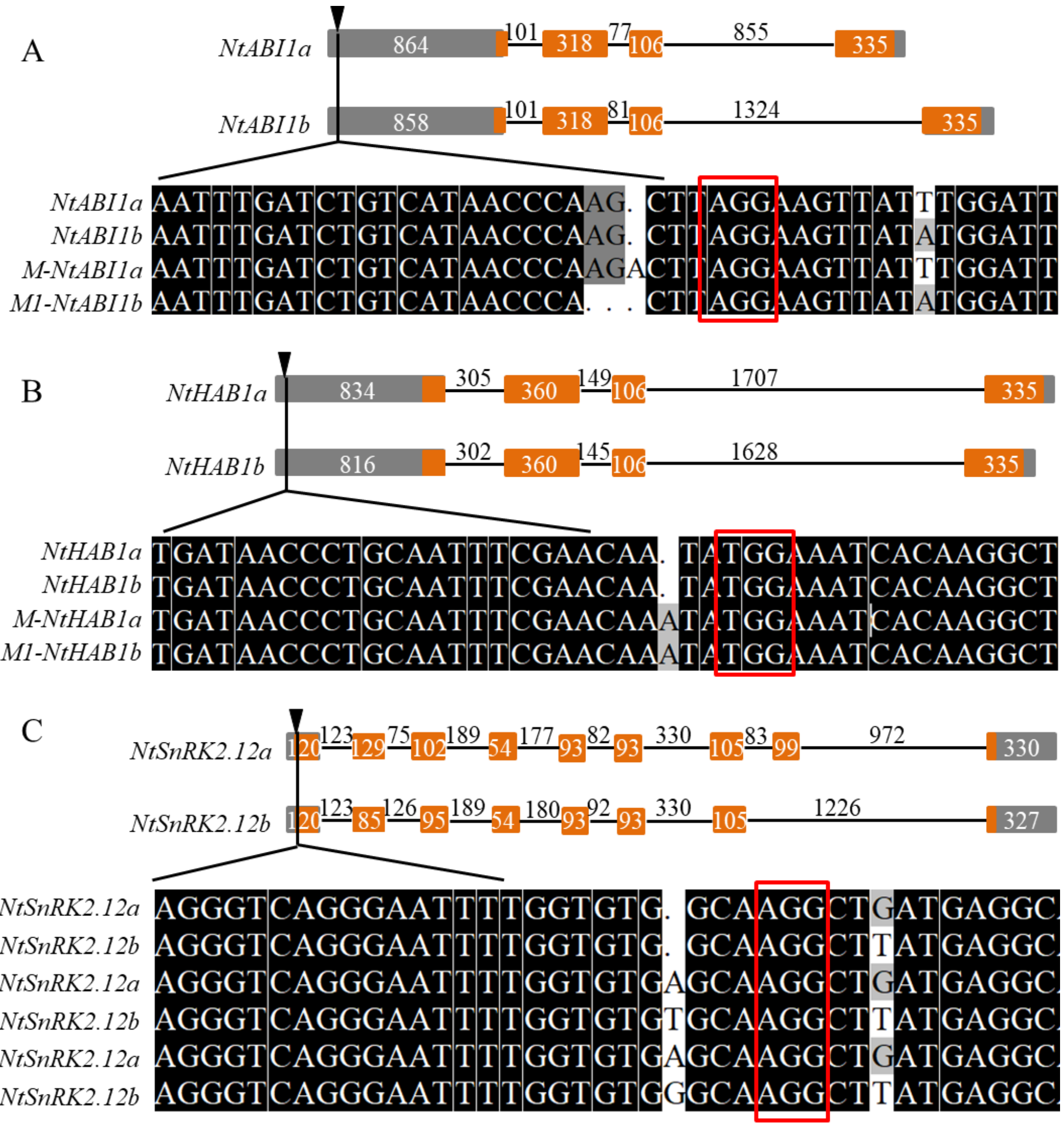

Supplementary Figure S10. Generation of *ntabi1*, *nthab1*, and *ntsnrk2.12* mutants by Crispr/cas9. A, Gene structures of *NtABI1a /1b* and identification of their knock-out mutant by Hi-TOM sequencing. B, Gene structures of *NtHAB1a /1b* and identification of their knock-out mutant by Hi-TOM sequencing. C, Gene structures of *NtSnRK2.12a /2.12b* and identification of their knock-out mutant by Hi-TOM sequencing. The numbers indicate the length (bp) of exons or introns. The orange boxes indicate the putative Pfam domains. The triangles show the location of sgRNA for Crispr/cas9. Red boxes indicate the PAMs.

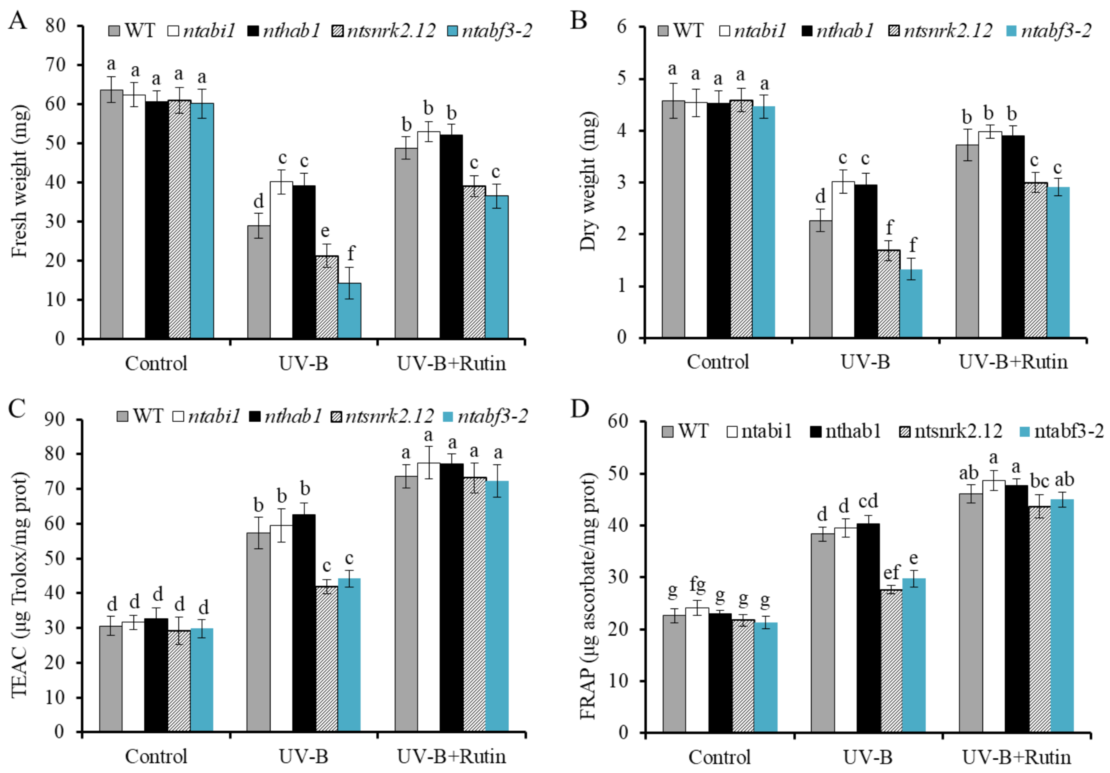

Supplementary Figure S11. Phenotypic characterization of different mutants under various treatments. A and B, Fresh and dry weight of the above ground parts of WT, *ntabi1*, *nthab1*, *ntsnrk2.12*, and *ntabf3-2* seedlings under different treatments. Values are means ±SD of ten independent replicates. C and D, Total antioxidant capacities (TEAC and FRAP) of the WT, *ntabi1*, *nthab1*, *ntsnrk2.12*, and *ntabf3-2* seedlings under different treatments. Values are means ±SD of five independent replicates. The significant differences are detected by Tukey's test ($p < 0.05$) and shown with letters.

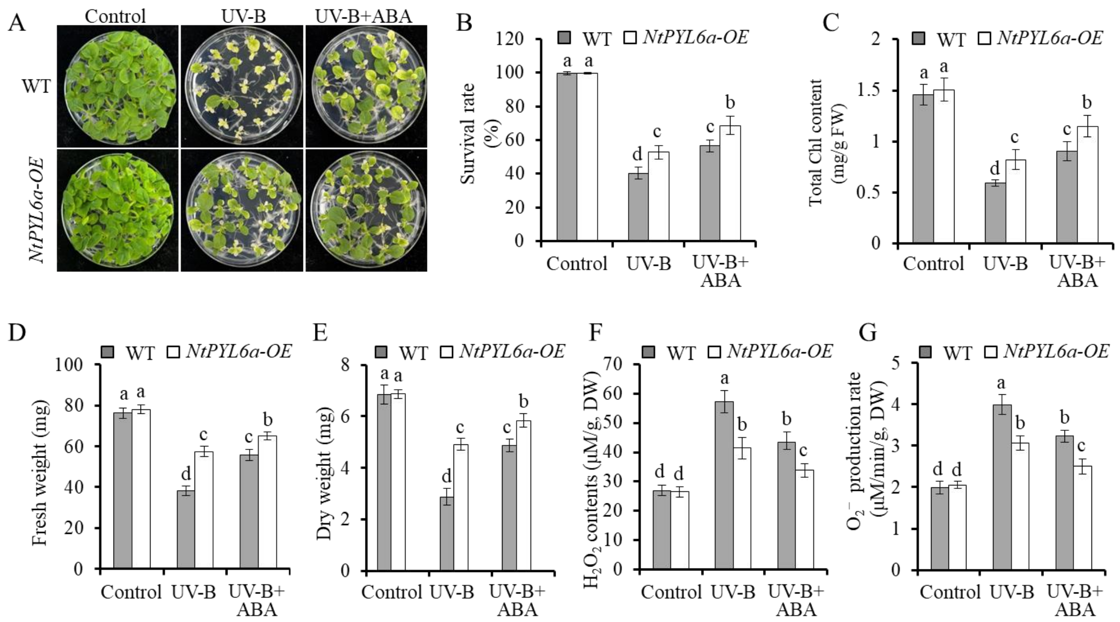

Supplementary Figure S12. Over-expression of *NtPYL6a* enhances plant resistance to UV-B. A, Phenotypes of WT and *NtPYL6a-OE* seedlings with or without UV-B treatment. B and C, Survival rates and total Chl contents of the control and UV-B treated WT and *NtPYL6a-OE* seedlings. Values are means ±SD of five independent replicates. The significant differences are detected by Tukey's test ($p < 0.05$) and shown with letters. D and E, Fresh and dry weight of the above ground parts of WT and *NtPYL6a-OE* seedlings. Values are means ±SD of five independent replicates. The significant differences are detected by Tukey's test ($p < 0.05$) and shown with letters. F and G, The contents of H_2O_2 and O_2^- in the WT and *NtPYL6a-OE* seedlings under different treatments. Values are means ±SD of ten independent replicates. The significant differences are detected by Tukey's test ($p < 0.05$) and shown with letters.

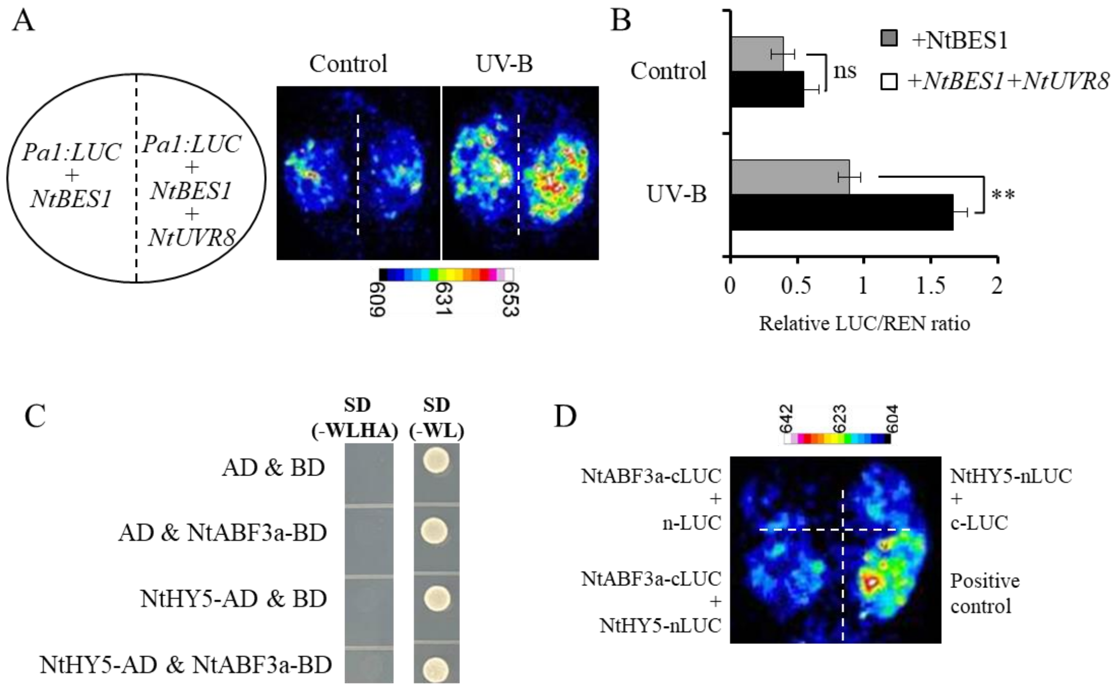

Supplementary Figure S13. Detection of protein-protein interactions and their effects. A and B, LUC intensities of *Pa1:LUC* co-expressed with different proteins. The color scale bar indicates the gray value. Values are means ±SD of three independent replicates. Asterisks indicate the significant differences, which are detected by two-tailed paired Student's t-test (**, $p < 0.01$). C, Y2H assay showed that there was no interaction between NtABF3a and NtHY5 in yeast. D, Split-LUC assay showed no LUC fluorescence between NtABF3a-cLUC and NtHY5-nLUC. The color scale bar indicates the gray value.
